# Supplementary material for: Reducing missed opportunities for vaccination in Mozambique: findings from a cross-sectional assessment conducted in 2017
Source: BMJ Open. 2021 Dec 30;11(12):e047297. doi: 10.1136/bmjopen-2020-047297 (PMC8718423; doi:10.1136/bmjopen-2020-047297)
Supplement: Supplementary data [file bmjopen-2020-047297supp001.pdf]

Supplementary Table 1. Immunization performance indicators among selected provinces and districts in Mozambique.

Province	District	Performance indicator*		
		Immunization coverage throughout province or district	Health facilities with functioning refrigerators	Health facilities experiencing a stockout of at least one antigen
Niassa	Mecanhelas	98%	78%	10%
	Majune	40%	50%	25%
Zambezia	Nicoadala	86%	85%	19%
	Gile	53%	50%	19%
Maputo Provincia	Manhica	83%	85%	13%
	Namahacha	35%	78%	19%

*Average for March through June 2017. (Source: Sistema Electrónico de Logística de Vacinas and District Health Information System 2 (SELV and DHIS2))

Supplementary Table 2: Time intervals used to classify timeliness of vaccination doses received using the nationally recommended ages for vaccination in Mozambique, 2017.

Vaccine	Recommended age of vaccination	Vaccination given; too early	Vaccination given; timely	Vaccination given: delayed
**Bacille Calmette-Guerin (BCG)**	At birth up to 23 months of age	--	0-45 days	>45 days
**Zero dose of OPV**	At birth or before 6 weeks of age	--	0-45 days	>45 days
**First dose of penta, OPV, PCV, and rotavirus**	At 2 months up to 23 months of age	<60 days	60-89 days	>89 days
**Second dose of penta, OPV, PCV, and rotavirus***	At 3 months or 4 weeks after first dose(s) up to 23 months of age*	<90 days	90-119 days	>119 days
**Third dose of penta, OPV, PCV, IPV and rotavirus***	At 4 months or 4 weeks after second dose(s) up to 23 months of age*	<120 days	120-149 days	>149 days
**First dose of measles**	9 months up to 23 months of age	<270 days	270-299 days	>299 days
**Second dose of measles and rubella**	18 months up to 23 months of age	<540 days	540-569 days	>569 days

Abbreviations: OPV, oral poliovirus vaccine; penta, diphtheria-tetanus-pertussis-hepatitis B–*Haemophilus influenzae* type b vaccine; IPV, inactivated poliovirus vaccine; PCV, pneumococcal conjugate vaccine.
* Rotavirus vaccine given up to 14 weeks of age.

Supplementary Table 3. Characteristics of surveyed caregivers of children with documented vaccination histories, Mozambique, 2017.

CAREGIVER SURVEY	n	%
	538	
Child demographics		
**Sex**		
Female	273	51
Male	265	49
**Age**		
<12 months	396	74
≥12 months	142	26
**Province**		
Maputo	193	36
Niassa	183	34
Zambezia	162	30
**Ever vaccinated**	525	
Yes	450	87
No	75	13
Caregiver demographics		
**Sex**	538	
Female	524	97
Male	14	3
**Relationship to child**	535	
Mother	508	95
Father	22	4
Uncle/aunt/grandparent	5	1
**Can read and write**	529	
Yes	236	45
No	293	55
**Educational Level**	532	
No educational qualifications	216	41
Did not complete primary education (less than 6 years)	108	20
Completed primary school	153	29
Completed secondary school or had higher education	55	10
Knowledge and attitudes		
**Child has home-based record**		
Yes, available at visit	533	99
Yes, but not available at visit	3	<1

No	2	<1
**Do you know precisely the vaccines that your child needs?**	515	
Yes	79	15
No	387	75
Not sure	49	10
**What is the purpose of a vaccines?***	535	
To prevent diseases	372	70
So children grow up healthy	219	41
To cure or heal diseases	28	5
They don't do any good	2	0
Not sure	112	21
Health facility visit		
**Type of health facility**	522	
Health center	500	96
Hospital	22	4
**Purpose of health facility visit**		
Medical consultation	145	27
Vaccination	143	27
Healthy child check-up	221	41
Accompanying caregiver	18	3
Child registration	7	1
Hospitalization	3	<1
**Health worker asked for the home-based record**	537	
Yes	464	86
No	73	14
**If vaccinated today, informed about next vaccination date?**	147	
Yes	113	77
No	34	23
**If vaccinated today, told which vaccines were given?**	147	
Yes	71	48
No	76	52
**If vaccinated today, told about vaccination reactions?**	147	
Yes	52	35
No	95	64
**If vaccinated today, satisfied with service?**	147	
Yes	141	96
No	6	4

**Suggestions for improving vaccination services***	530	
There should be more vaccination staff	120	23
There should be less waiting time	111	21
More information about vaccines given, diseases vaccines prevent, and AEFIs	93	18
There should always be vaccines in stock and functioning fridge	78	15
Friendlier treatment of the public	58	11
No suggestions or not sure	223	42

*Respondents allowed to select multiple responses.

Supplementary Table 4. Characteristics and knowledge, attitudes, and practices of surveyed health workers, Mozambique, 2017.

HEALTH WORKER SURVEY	n	%
	223	
Health worker demographics		
**Sex**	222	
Female	120	54
Male	102	46
**Professional training**	222	
Doctor	5	2
Nurse	91	41
Public employee in the health sector	9	4
Other	117	53
**Years of experience**	219	
0 to 4	144	66
5 to 9	66	30
10+	9	3
**Ever trained in vaccination or vaccine-preventable diseases**	221	
Yes	183	83
No	38	17
Health worker knowledge, attitudes, practices		
**My knowledge of vaccination and the EPI is sufficient to meet its needs**	222	
Agree	174	78
Disagree	48	22
**Contraindications for any vaccine***	219	
Local reaction to previous dose	60	27
Low grade fever	95	43
Seizures under medical treatment	142	65
Pneumonia and other serious diseases	71	32
None of the above	26	12
**When should vaccination status be assessed?***	222	
During child welfare visit	72	32
Consultation for any illness	61	27
When a child is accompanying a caregiver during a prenatal examination	17	8
When a child is accompanying a caregiver who visits a health centre for any reason	28	13

All of the above	112	50
**Why is vaccination status incomplete for some children?***	222	
Parents' negative beliefs related to vaccination	124	56
Hours of vaccination are incompatible with schedule of parents	28	13
Health workers do not review children's vaccination cards or about vaccination status	24	11
False contraindications for vaccination by health workers	36	16
Distance from vaccination site	174	78
All of the above	16	7
**I fear adverse reactions to vaccines**	220	
Agree	158	72
Disagree	62	28
**Completing nominal registers delays timely vaccination**	221	
Agree	44	20
Disagree	177	80
**There is sufficient staff offering immunization services at this facility**	49	
Agree	33	67
Disagree	16	33
**Enough vials of vaccine for all patients in need**	49	
Agree	38	78
Disagree	11	22

*Respondents allowed to select multiple responses.
