# Supplementary material for: Reducing missed opportunities for vaccination in Mozambique: findings from a cross-sectional assessment conducted in 2017
Source: BMJ Open. 2021 Dec 30;11(12):e047297. doi: 10.1136/bmjopen-2020-047297 (PMC8718423; doi:10.1136/bmjopen-2020-047297)
Supplement: Author's manuscript [file bmjopen-2020-047297.draft_revisions.pdf]

BMJ Open is committed to open peer review. As part of this commitment we make the peer review history of every article we publish publicly available.

When an article is published we post the peer reviewers' comments and the authors' responses online. We also post the versions of the paper that were used during peer review. These are the versions that the peer review comments apply to.

The versions of the paper that follow are the versions that were submitted during the peer review process. They are not the versions of record or the final published versions. They should not be cited or distributed as the published version of this manuscript.

BMJ Open is an open access journal and the full, final, typeset and author-corrected version of record of the manuscript is available on our site with no access controls, subscription charges or pay-per-view fees (http://bmjopen.bmj.com).

If you have any questions on BMJ Open's open peer review process please email info.bmjopen@bmj.com

BMJ Open

Reducing missed opportunities for vaccination (MOV) in Mozambique: Findings from a cross-sectional MOV assessment conducted in 2017

Journal:	*BMJ Open*
Manuscript ID	bmjopen-2020-047297
Article Type:	Original research
Date Submitted by the Author:	26-Nov-2020
Complete List of Authors:	Magadzire, Bvudzai; VillageReach, Health Systems<br>Joao, Gabriel<br>Bechtel, Ruth<br>Matsinhe, Graça; Ministra da Saude<br>Nic Lochlainn, Laura; World Health Organization<br>Ogbuanu, Ikechukwu Udo; World Health Organization
Keywords:	PUBLIC HEALTH, Paediatric infectious disease & immunisation < PAEDIATRICS, International health services < HEALTH SERVICES ADMINISTRATION & MANAGEMENT, PRIMARY CARE

SCHOLARONE™
Manuscripts

I, the Submitting Author has the right to grant and does grant on behalf of all authors of the Work (as defined in the below author licence), an exclusive licence and/or a non-exclusive licence for contributions from authors who are: i) UK Crown employees; ii) where BMJ has agreed a CC-BY licence shall apply, and/or iii) in accordance with the terms applicable for US Federal Government officers or employees acting as part of their official duties; on a worldwide, perpetual, irrevocable, royalty-free basis to BMJ Publishing Group Ltd ("BMJ") its licensees and where the relevant Journal is co-owned by BMJ to the co-owners of the Journal, to publish the Work in this journal and any other BMJ products and to exploit all rights, as set out in our licence.

The Submitting Author accepts and understands that any supply made under these terms is made by BMJ to the Submitting Author unless you are acting as an employee on behalf of your employer or a postgraduate student of an affiliated institution which is paying any applicable article publishing charge ("APC") for Open Access articles. Where the Submitting Author wishes to make the Work available on an Open Access basis (and intends to pay the relevant APC), the terms of reuse of such Open Access shall be governed by a Creative Commons licence – details of these licences and which Creative Commons licence will apply to this Work are set out in our licence referred to above.

Other than as permitted in any relevant BMJ Author's Self Archiving Policies, I confirm this Work has not been accepted for publication elsewhere, is not being considered for publication elsewhere and does not duplicate material already published. I confirm all authors consent to publication of this Work and authorise the granting of this licence.

For peer review only

Title: Reducing missed opportunities for vaccination (MOV) in Mozambique: Findings from a cross-sectional MOV assessment conducted in 2017

Short title: Reducing missed opportunities for vaccination in Mozambique

Authors:

Bvudzai Priscilla MAGADZIRE*, VillageReach (bvudzai.magadzire@villagereach.org), 2900 Eastlake Ave. E, Suite 230, Seattle, Washington 98102, USA

Gabriel JOAO, VillageReach (gabriel.joao@villagereach.org)

Ruth BECHTEL, VillageReach (ruth.bechtel@villagereach.org)

Graca MATSINHE, Ministério da Saúde de Moçambique (gmatsinhe@gmail.com)

Laura NIC LOCHLAINN, World Health Organization, Headquarters, Geneva, Switzerland (niclochlainnl@who.int)

Ikechukwu Udo OGBUANU, World Health Organization, Headquarters, Geneva, Switzerland (dr_iyke@yahoo.com; ogbuanui@who.int)

*Corresponding author

Keywords: missed opportunities for vaccination; immunization; vaccination coverage; health care utilization; Mozambique

For peer review only

ABSTRACT

Objective: Factors that contribute towards poor vaccine coverage are often poorly understood. However, many missed children may already be accessing treatment and vaccination services, indicating that Missed Opportunities for Vaccination (MOV) are a significant contributing factor to under-vaccination. To better understand the magnitude and underlying causes for MOV among children (0-23 months), we conducted an assessment in 2017 and developed a roadmap to strengthen the immunization programme in Mozambique.

Design: Cross-sectional

Setting: Three provinces in North, South and Central Mozambique.

Methods: The assessment applied a mixed-methods approach. From a sample of 41 health facilities, we conducted exit interviews with caregivers of children 0-23 months (N=546); surveys with health workers (N=223); focus group discussions with caregivers (n=6) and health workers (n=5); and in-depth interviews with facility managers (n=9). We analyzed data to assess the magnitude of MOV and to identify reasons for MOV and factors that could inform interventions to prevent MOV.

Results: Of the 538 children with vaccination documentation, 60% (n=324) were eligible for vaccination at the start of the visit. Of these, 76% (n=245) had a MOV. Our analysis revealed that reasons for MOV were related to caregiver, health worker and health systems reasons. Inadequate information about vaccination among both caregivers and health workers, poor or poorly understood health facility practices, lack of process integration between curative and preventative services, inefficient tracking systems to support identification of children due for vaccination, and to a limited extent, poor supply of vaccines and other injection supplies at service points led to MOV.

Conclusions: The results of the assessment informed the development of roadmaps to reduce MOV. These may be applicable to other contexts. The global immunization community should continue to invest in efforts to reduce MOV, as it prioritizes making more efficient use of existing health service visits.

For peer review only

Strengths and limitations of this study

- The Ministry of Health teams were able to utilize the results immediately to inform the development of roadmaps to reduce MOV.
- The assessment covered a relatively short time period (10 to 12 days), in a small sample of health facilities and only included children with vaccination documentation, therefore other reasons for MOV may have been missed.
- As with past MOV assessments, this assessment was not intended to be nationally representative or representative of each of the three provinces that were sampled therefore results may not be generalizable.
- The estimation of MOV was limited only to children with documented vaccination dates extracted from children's home-based records or from health facility registers. We noted poor vaccination recording practices which could have resulted in many children being under- or over-vaccinated with certain antigens, leading to over- or under-estimation of MOV.

For peer review only

INTRODUCTION

Globally, the proportion of children receiving three diphtheria-tetanus-pertussis (DTP3) routine immunizations is below the global target of 90%, and has remained around 85% since 2015.(1) Although several factors may account for this, persistently high rates of missed opportunities for vaccination (MOV), particularly in low- and middle-income countries, are a significant contributing factor.(2) A MOV is defined by the World Health Organization (WHO) as "any contact with health services by an individual who is eligible for vaccination, which does not result in them receiving all the vaccine doses for which he or she is eligible."(3-5) It is widely assumed that children who are consistently missed by national immunization programmes, sometimes referred to as the "fifth child," have limited access to health services. However, evidence suggests that a significant proportion of the missed children, may already be accessing treatment and even seeking vaccination services — but still not receiving all of the recommended routine vaccinations.(3-5)

There is increasing momentum at global, regional, and country levels to address factors that may result in MOV. In response to this, the WHO has been working with multiple immunization partners since 2015 to update and scale up the global MOV strategy. The MOV strategy, which consists of four main components: the *WHO Planning Guide to Reduce Missed Opportunities for Vaccination (MOV)*] (4), the revised and updated *Methodology for the Assessment of Missed Opportunities for Vaccination* (5), the *Intervention Guidebook for Reducing Missed Opportunities for Vaccination (MOV)* (6), and coordination

For peer review only - http://bmjopen.bmj.com/site/about/guidelines.xhtml

among immunization partners and countries.

The MOV strategy aims to answers three important questions:

- How many opportunities for vaccination are missed at existing vaccination sites?
- Why are opportunities for vaccination missed at these vaccination sites?
- What can be adjusted or done differently (e.g., changes in policies, changes in behaviours, or structural or organizational changes) to reduce MOV?

By identifying the underlying causes for MOV, countries can tailor health system interventions to reduce MOV, with the goal of improving vaccine coverage, timeliness, and equity.

The Mozambique Expanded Programme on Immunization (EPI) has experienced challenges with increasing immunization coverage, with national coverage of DTP3 and the first dose of measles containing vaccine (MCV1) remaining at 80%, and 85%, respectively since 2015.(7) At the subnational level, many areas of the country have varying coverage levels and have received varying degrees of vaccine delivery strengthening interventions from optimized vaccine supply chains to short-term interventions, such as immunization campaigns. (8, 9) Despite these multi-stakeholder efforts to address challenges, new cases of circulating vaccine-derived poliovirus (cVDPV) and pockets of unvaccinated children in Mozambique indicate an urgency to strengthen the EPI. (10)

To address these challenges, in 2017, the Ministry of Health (MoH) along with a consortium of partners, including WHO, and VillageReach (a non-profit global health organization), implemented the MOV strategy in Mozambique.(4-6) The objectives were to estimate the prevalence of MOV, timeliness of vaccination, factors associated with MOV among children aged 0-23 months, health worker knowledge, attitude and practices, and to document the resulting recommendations to improve immunization policies and practices in Mozambique.

METHODS

Study design and setting

The MOV assessment methodology uses a mixed-methods approach incorporating both quantitative and qualitative tools. Quantitative data were collected by conducting exit interviews with caregivers and knowledge, attitudes, and practices (KAP) surveys with health workers. Qualitative data were collected through focus group discussions (FGDs) with caregivers and health workers, as well as through in-depth

For peer review only - http://bmjopen.bmj.com/site/about/guidelines.xhtml

interviews (IDIs) with health facility managers. The detailed MOV assessment methodology has been described elsewhere. (1, 11-15)

The MoH selected the following three provinces: Niassa (Northern region), Zambezia (Central region) and Maputo (South) for geographic representation and also due to the presence of VillageReach in these regions. In each province, a further two districts were selected. For district selection, we conducted a desk review using two electronic record systems as data sources. One was an electronic system for vaccine logistics (SELV [Sistema Electrónico de Logística de Vacinas) and the second was a monitoring and evaluation information system (SIS-MA [Sistema de Informação de Saúde para Monitoria e Avaliação]). We reviewed data for a period of three months (April to June 2017), examining the following criteria: administrative vaccination coverage, vaccine stock-out rates, and functioning of cold chain equipment (Supplementary table 1). Districts with vaccination coverage greater than 80%, a stock-out rate lower than 10%, and more than 95% of fridges functioning were considered "well-performing." Districts with vaccination coverage less than 50%, a stock-out rate higher than 10%, and fewer than 95% of fridges functioning were considered "poor performing."

Within each province a combination of comprehensive primary health care and tertiary facilities that serve populations of different socioeconomic and vulnerability status in urban and rural areas were purposively selected.

Participant selection

Health facilities staff is made up of a mix of mostly nurses and technicians and a few doctors. For the health worker KAP surveys and FGDs, we included health workers in the selected health facilities from both preventative and curative departments who were present on the day of the assessment.

Patient and public involvement

Caregivers collaborated with us as key informants. Their views and experiences alongside those of health workers shaped the design of proposed interventions. All caregivers aged at least 18 years old exiting the health facility in the selected districts and with a child that appeared to be 0 through 23 months were eligible for inclusion in the exit surveys or FGDs. If more than one child accompanied a caregiver, the interviewers were instructed to select one child that was 0 through 23 months of age. (5)

Data collection tools

The generic data collection tools in the WHO MOV methodology guide were translated into Portuguese.(5) All quantitative data were collected on password protected tablets using survey forms

For peer review only - http://bmjopen.bmj.com/site/about/guidelines.xhtml

uploaded to the survey software platform (Zegeba AS [Alesund, Norway]). The data collectors directly entered data from vaccination documentation (home-based records (HBRs) or health facility registers) into the survey platform software and a photo of the vaccination documentation was captured for data validation purposes. Qualitative data notes were recorded on paper, and subsequently typed up and emailed to the MOV supervision team. Paper notes were destroyed once backed up electronically and were then translated into English for analysis.

Data collection and analysis

Prior to data collection, we conducted a week-long training with the field team, which comprised of nine supervisors and data collectors. The field team included EPI staff from the MoH, independent researchers from Eduardo Mondlane University, and VillageReach staff. To consolidate classroom training lessons and ensure comfort with electronic data entry of the surveys and taking photos of vaccination documentation, we piloted the data collection tools in nearby health facilities in Maputo city. Mozambique was the first country to use the Portuguese version of the WHO MOV tools; therefore, after the pilot, the team assessed the accuracy of the translation and amended the tools as necessary.

Field teams were deployed to the three provinces over a two-week period between October and November 2017. The teams aimed to achieve a daily team quota of 20 exit interviews with caregivers and 10 KAP surveys with health workers at each health facility. Exit interviews with caregivers were conducted in the morning, and health workers were interviewed in the afternoon. Health facility and sub-national managers were purposively selected to participate in in-depth interviews. Although an initial sample of 30 health facilities were selected centrally, team supervisors liaised with the central coordinator to add new sites if the session sizes at the health facility was insufficient to fulfil the daily team quota. Because data were captured primarily with electronic tools (tablets), quantitative data were uploaded daily to a central server and translated to English for analysis.

All quantitative analyses were conducted using Stata (StataCorp. 2015. Stata Statistical Software: Release 14. College Station, TX: StataCorp LP). Data analysis focused on estimating the extent of MOV using information collected from vaccination documentation. As part of the data cleaning process, all vaccination dates entered by the field teams during data collection were validated by reviewing each photo of the vaccination documentation. We further analyzed other explanatory and demographic factors for reasons for MOV, the classification of MOV by underlying cause (e.g., health system-, caregiver-, or health worker–related) and estimated the number of eligible doses missed, by antigen. We assessed the timeliness of vaccination doses using the recorded date of birth, the date of

For peer review only - http://bmjopen.bmj.com/site/about/guidelines.xhtml

vaccination from vaccination documentation, and the Mozambique EPI-recommended time intervals for vaccines (Supplementary Table 2).

Using Atlas.ti (ATLAS.ti Scientific Software Development GmbH, version 8), we conducted a thematic analysis of the qualitative data transcripts to explore common, unique, and conflicting themes, as well as to draw out illustrative quotes and key conclusions.

As part of the results harmonization process, we shared the preliminary MOV assessment results with the leadership of the three provinces for fact checking and to inform the development of future interventions to reduce MOV.

For peer review only

Ethics approval

The National Bioethics Committee in Mozambique (Nacional de Bioetica) granted ethical clearance for this assessment (Reference Number: 1008). In addition, each province (Niassa, Zambezia, and Maputo) granted permission for the MOV assessment to be conducted. Before the interview, respondents were informed that participation was voluntary and optional. They were also informed that their responses will in no way affect their ability to access services or, in the case of health workers, threaten their employment.

RESULTS

Respondent characteristics

The field teams conducted the MOV assessment in 41 health facilities, visiting an additional 11 health facilities than planned, due to small session sizes encountered in some health facilities. The teams carried out exit interviews with 546 caregivers, of which 98% (n=538) had children aged 0-23 months and 95% (n=521) had vaccination documentation. For 41% (n=221) of caregivers, the purpose of health facility visit was for a healthy child check-up, followed by 27% (n=145) for medical consultation; 27% (n=143) for vaccination; 3% (n=18) accompanying caregiver; 1% for child registration (n=7) or hospitalization (n=3) (Supplementary Table 3).

In terms of age, three-quarters (74%; n= 396) of the children were younger than 12 months and 51% (n=273) were female (Supplementary Table 3). The caregiver literacy rate (measured as reported ability to read and write) was 45% (n=236). Forty-one percent (n=216) of caregivers had no educational qualifications, while 29% (n=153) had completed primary education, and 10% (n=55) had completed secondary school or had higher education (Supplementary Table 3).

For peer review only - http://bmjopen.bmj.com/site/about/guidelines.xhtml

We also conducted KAP surveys with 223 health workers at the selected health facilities, including staff from preventative and curative service departments. The majority of health workers interviewed were trained nurses (42%; n=93), doctors (34%; n=75) and the remainder were other professional cadres from pharmacist/pharmacist technician (7%; n=16) to laboratory technician (4%; n=8). Among participating health workers across the three provinces, only 16% (n=49) routinely administer vaccines in the EPI clinic. The remainder worked in outpatient departments, oral health, general maternal and child health, family planning, nutrition, or another specialty (data not shown). Two-thirds (n=144) of health workers had less than four years work experience. Most health workers (83%; n=183) reported they received training in vaccine-preventable diseases during basic pre-service professional training (Supplementary Table 4). However, only 22% (n=48) had received additional in-service training. Of those who had received in-service training, 53% (n=24) had been trained in the past year, 20% (n=9) in the past two to three years, and 27% (n=12) were trained four or more years ago.

Finally, the field teams conducted six FGD sessions with caregivers, five FGD sessions with health workers, and nine in-depth interviews with sub-national and health facility managers.

Frequency and causes of MOV

Proportion of children with MOV

The proportion of children whose vaccinations were up to date at the start of the visit was 40% (n=214), among which, one child was not eligible for vaccination, as they had a reaction to a previous vaccine dose. Among the 324 children eligible for vaccination, 76% (n=245) had a MOV on the day of the assessment. Among the 324 children, 52% (n=170) did not receive any vaccine, while 23% (n=75) received at least one dose during the visit, but were still due for other vaccines, and 24% (n=79) received all due vaccines (Figure 1).

For peer review only

Demographic and other factors correlated with MOV

We found the proportion of MOV differed slightly by sex, with 76% (n=118) of females compared to 70% (n=117) of males experiencing a MOV. Children aged 12 months or older had a higher proportion of MOV (95%; n=58), compared to children <12 months (67%; n=177). Children who lived in Zambezia Province had a higher proportion of MOV (93%; n=98) compared to children from Maputo (76%; n=74) and Niassa (56%; n=61).

Forty-five percent (n=59) of children eligible for vaccination and who were at the health facility for vaccination had a MOV, compared with 91% (n=176) of children whose reason was for a non-vaccination visit. Among these children, 93% (n=69) had a MOV during their medical consultations, 89% (n=92) during a healthy child visit and 92% (n=12) when accompanying a caregiver.

When caregivers were asked why their child was not vaccinated during the health facility visit, 59% (n=92) said that the reason for their health facility visit was not for vaccination or they believed their child was already fully vaccinated (18%; n=29). Caregivers also said that (10%; n=15) of health workers told them their child was not eligible for vaccination or did not tell them about vaccination during the visit 8% (n=12) or because there were no vaccines available in the health facility (8%; n=12) (Table 1).

For peer review only

Table 1. Numbers and proportion of missed opportunities for vaccination (MOV) in three provinces in Mozambique, by demographic and other factors

Demographic or other factor	Overall: No contraindications, and due at least 1 dose n (%)	Overall: MOV n (%)
	324 (60%)	245 (76%)
Sex of child		
Female	156 (48%)	118 (76%)
Male	168 (52%)	117 (70%)
Age of child		
<12 months	263 (81%)	177 (67%)
≥12 months	61 (19%)	58 (95%)
Province		
Maputo	102 (31%)	76 (74%)
Niassa	114 (35%)	61 (56%)
Zambezia	105 (33%)	98 (93%)
Reason for visit		
Vaccination	130 (40%)	59 (45%)
Non-vaccination visit	194 (60%)	176 (91)
i. Healthy child visit	*103 (32%)*	*92 (89%)*
ii. Medical consultation	*74 (23%)*	*69 (93%)*
iii. Accompanying caregiver	*12 (4%)*	*11 (92%)*
iv. Hospitalization	*2 (<1%)*	*2 (100%)*
v. Child registration	*2 (<1%)*	*2 (100%)*
Do you have a home-based record for this child?		
Yes, and I have it with me today	319 (98%)	230 (72%)
Yes, but I do not have it with me	3 (1%)	3 (100%)
No, I do not have a home-based record for this child	2 (1%)	2 (100%)
Reason not vaccinated today*		
Vaccination was not reason for visit	-	92 (59%)
Child is already fully vaccinated	-	29 (18%)
Health worker said child was not eligible for vaccination	-	15 (10%)
No vaccines in the health facility	-	12 (8%)
Health worker did not tell me about vaccinating child	-	6 (4%)
Other reasons	-	3 (2%)

**Respondents allowed to select multiple responses.*

Eligible vaccine doses missed, by antigen

Of the 851 eligible vaccine doses due, 53% were missed (n=447). Bacille Calmette-Guérin (BCG) (60%; n=40), first dose of oral poliovirus (OPV1) (64%; n=39), third dose of OPV (OPV3) (66%; n=38), second dose of measles and rubella (89%; n=33) and inactivated poliovirus (IPV) vaccine (91%; n=117) constituted the highest number of missed doses overall (Table 2).

Table 2. Missed opportunities by vaccine dose and timeliness of vaccine doses administered to surveyed children 0-23 months with documented vaccination history, Mozambique 2017

Vaccine and dose	Eligible doses due n	Eligible due doses		Timeliness[1]			
		Given n (%)	Missed n (%)	Doses children received n	Vaccination given; Too early %	Vaccination given; Timely %	Vaccination given; Delayed %
Bacille Calmette-Guerin (BCG)	67	27 (40%)	40 (60%)	498	—	90%	10%
Zero dose OPV	32	20 (62%)	12 (38%)	461	—	97%	3%
First dose of Penta	47	31 (66%)	16 (34%)	459	12%	76%	12%

For peer review only

First dose of OPV	61	22 (36%)	39 (64%)	436	12%	71%	17%
First dose of PCV	48	19 (60%)	29 (40%)	456	11%	76%	13%
First dose of rotavirus*	38	28 (74%)	10 (26%)	419	11%	75%	14%
Second dose of Penta	47	39 (83%)	8 (17%)	417	9%	75%	16%
Second dose of OPV	45	25 (56%)	20 (44%)	385	9%	66%	25%
Second dose of PCV	48	38 (79%)	10 (21%)	411	9%	71%	20%
Second dose of rotavirus*	44	40 (91%)	4 (9%)	368	9%	50%	41%
Third dose of Penta	49	25 (51%)	24 (49%)	350	6%	73%	21%
Third dose of OPV	58	20 (34%)	38 (66%)	321	6%	64%	29%
Third dose of PCV	55	26 (47%)	29 (53%)	340	6%	70%	24%
Inactivated poliovirus	129	12 (9%)	117 (91%)	274	16%	65%	18%
First dose of measles	46	28 (61%)	18 (39%)	216	14%	67%	19%
Second dose of measles and rubella	37	4 (11%)	33 (89%)	23	4%	39%	57%
**Total**	**851**	404 (47%)	447 (53%)	**5,834**	**10%**	**70%**	**21%**

Note: [1] Please see Supplementary Table 2 for time intervals used for classifying timeliness of vaccination as per the Mozambique immunization schedule. An em dash (—) indicates that no eligible doses were due. *Rotavirus vaccine given up to 14 weeks of age.
Abbreviations: OPV, oral poliovirus vaccine; penta, diphtheria-tetanus-pertussis-hepatitis B–*Haemophilus influenzae* type b vaccine; IPV, inactivated poliovirus vaccine; PCV, pneumococcal conjugate vaccine.

Timeliness of routine immunization

Overall, 70% (n=4,102) of the 5,834 documented vaccine doses were administered in a timely manner, while 10% (n=558) were too early and 21% (n=1,236) were delayed. The vaccine doses most commonly administered too early were IPV (16%; n=45) and first dose of measles (14%; n=31) (Table 2). The most frequent delayed vaccines included 41% (n=151) of second dose of rotavirus, 25% (n=98) of second dose of OPV, 30% (n=94) of third dose of oral poliovirus, and 57% (n=13) of measles-rubella second dose. Overall, the pentavalent and OPV vaccine series both showed decreasing timeliness, with doses that were due at older ages tending to be more delayed (Table 2).

We also found 13 children with more than one dose of IPV, and 37 children with a third dose of rotavirus recorded in their HBR (Supplementary Table 2).

Quality, availability, and accessibility of vaccines and vaccination services

During the health facility visit, 86% (n=464) of caregivers reported that the health worker asked to see their child's HBR. Among the 154 caregivers who brought their child for vaccination on the day of the assessment, 96% (n=141) reported being satisfied with the services. However, 64% (n=95) were not told about vaccination reactions their child might have following vaccination, 52% (n=76) were not told what vaccines their child had been given, and 23% (n=32) were not informed about the next vaccination date

For peer review only - http://bmjopen.bmj.com/site/about/guidelines.xhtml

(Supplementary Table 3).

Two-thirds (67%; n=33) of health workers who routinely administer vaccines in the EPI clinic felt there were sufficient staff to carry out vaccination activities. However, 22% (n=11) reported having insufficient supplies of specific vaccines including for BCG, pneumococcal conjugate, rotavirus, and oral poliovirus vaccines (Supplementary Table 4). Other supplies that were reported to be lacking at the service delivery level included syringes, HBRs, and safety boxes.

Physical accessibility (distances and geographic factors) to vaccination services was one of the major barriers identified by both caregivers and health workers in FGDs and in-depth interviews. Some caregivers stated that this is particularly challenging when the child is still very young, and they do not want to expose them to harsh weather conditions or when they do not have transportation to the health facility:

> "*What happens sometimes is that mothers do not appear…when it (health facility) is far away, they begin to have fear. Maybe they just come for those first vaccines until the (baby is) 4 months*". (Health worker FGD, Zambezia province)

Health workers cited mobile brigades (outreach teams) as playing an important role in addressing this and other issues, by bringing vaccination services to the communities. However, resources for mobile brigades in terms of transportation and human resources were limited. As a result, health workers said, maintaining a consistent outreach programme was challenging:

> *"Another challenge is to cover the entire area of the administrative post including the absentees. There are many absentees, they may give the contact number but it does not go through. Although some give their addresses, how do we get there? It's hard. Here many times, we plan mobile brigades but we can't run them because of insufficient transportation. We only have [an] ambulance and it is doing a lot of work hours and patient transfers"*. (Health worker FGD, Maputo province)

Knowledge and perceptions of vaccination among caregivers

Although a high percentage (99%; n=533) of caregivers with children 0-23 months of age had their children's HBR on hand during the health facility visit, the majority of caregivers (75%; n=387) reported not knowing precisely what vaccines their child needed, and 21% (n=112) were unsure about the purpose of vaccination (Supplementary Table 3). Of the 78 caregivers who reported knowing the vaccines needed by their children, 36% (n=28) did not know when the vaccines should be given. Gaps in

For peer review only - http://bmjopen.bmj.com/site/about/guidelines.xhtml

caregiver knowledge on issues related to vaccination were also echoed strongly in FGDs:

> *"… we don't know all the vaccines. We know that they protect, yes, but each one protects what, most of us don't know. Maybe an explanation in terms of what each of the vaccines do [would help]. And when they increase the vaccines, also explain to us why they are increasing and which vaccines they are adding".* (Caregiver, FGD, Niassa province)

Overall, caregiver responses seemed to reflect that they placed high value on vaccination services; 92% (n=470) stated that children can get sick if they are not vaccinated, and 97% (n=507) reported never having lost a child's HBR. However, caregivers also raised concerns during FGDs on vaccine acceptability. Caregivers mentioned that the number of vaccinations that children receive is too high:

> *"The big problem is also the amount of vaccines. Too many vaccines at the same time. There should be a way to reduce the vaccines because children suffer. You see that a child of nine months get four vaccines in a single day. That's a lot".* (Caregiver, FGD, Maputo province)

Knowledge and perception of vaccination among health workers

Seventy-eight percent (n=174) of health workers agreed that their knowledge about vaccines was outdated. A high percentage reported low-grade fever (43%; n=95) and seizures under medical treatment (63%; n=142) as contraindications for any vaccine. Seventy-two percent (n=152) of health workers also reported that they feared adverse events following immunization (AEFI). Only 63% (n=140) of health workers correctly selected all the vaccines that healthy children should receive. Fifty percent (n=112) of health workers selected that a child's vaccination status should be checked during every type of health service encounter (Supplementary Table 4). More than one-third (37%; n=51) suggested that only the health professional responsible for vaccination has an obligation to ask about vaccination status.

When health workers were asked why vaccination is incomplete for some children, 56% (n=124) reported that caregivers' negative perceptions of vaccination are a major contributing factor to children missing vaccinations.

Although 80% (n=177) of health workers did not perceive recording vaccine doses on health facility registers and HBR as a cause of delayed vaccination (Supplementary Table 4), during FGDs with health workers they said that they needed improved systems that would allow them to retrieve children's records quickly and follow-up with children who did not return for vaccine doses:

> *"… right now what we consider a challenge, is actually the children's retention in the vaccination programme, from the first to the last shot. We have books that allow us to follow*

For peer review only - http://bmjopen.bmj.com/site/about/guidelines.xhtml

the child from day one in the same book. Now, the search for defaulters is one of the greatest challenges". (health worker, FGD, Maputo province)

Health workers identified three actions as critical for follow-up of defaulting children, including home visits, developing a weekly list of children with incomplete programmes, and contacting caregivers via phone or other means of communication. One of the three provinces was already implementing these interventions, but reported needing more resources to improve the effectiveness of the intervention.

DISCUSSION

In this first assessment in Mozambique to assess MOV, we have shown that among 60% of children eligible and due for vaccinations at the start of the health facility visit, 76% had a MOV. Although 99% of caregivers had vaccination documentation available during the visit, which was the highest observed in a MOV assessment to date, nine in ten children (93%) who were at the health facility for non-vaccination visit had a MOV. We found the proportion of MOV was higher among females (76%) compared to males (70%) and 95% of children aged 12-24 months eligible for vaccination had a MOV. Nine in ten children (93%) who lived in Zambezia Province had a MOV compared to 74% of children in Maputo and 56% of children in Niassa.

Evidence from a growing body of WHO-supported MOV assessments suggests a significant proportion of children with access to health services experience MOV. (3, 11) We found that health services continue to miss the opportunity to vaccinate eligible children for a variety of reasons, even when these children access our health facilities for services. In addition, almost three-thirds of caregivers whose child had a MOV said that the reason for the health facility visit was not for vaccination. This provides evidence that there is insufficient screening and referral of children eligible for vaccination, highlighting a need for improved integration between preventative and curative services. The other most significant factors for missing eligible children included lack of inadequate systems for and practice of tracking children's vaccination status at the health facility level, inadequate caregiver knowledge and in fewer circumstances, due to vaccine stock-outs or refusal for coming on the wrong day.

Having a vaccination schedule that includes vaccines in the second year of life can often lead to a reduction in MOV, as it provides another opportunity for a child to receive catch-up vaccinations. In 2015, Mozambique introduced a second dose of measles-rubella at 18 months. (16) However, we found that almost all children aged 12-24 months eligible for vaccination had a MOV. In order to benefit from

For peer review only - http://bmjopen.bmj.com/site/about/guidelines.xhtml

this contact point, the delivery platform for vaccines in the second year of life must be further strengthened in Mozambique.

During brainstorming and debrief sessions at the province and national levels with EPI programme staff and immunization stakeholders, the key results were linked with actionable steps to reduce MOV and improve the EPI programme overall. Stakeholders deliberated on the interventions that were, in their view, feasible in the short term. Provinces drafted action plans for reducing MOV as part of the 2019 EPI plans. Some of the themes that emerged during the brainstorming sessions are discussed below. However, it must be noted that implementation of these plans were affected by Cyclone Ida and Cyclone Kenneth in early 2019, which had devastating consequences in Mozambique(17)

Having available supplies in health facilities is necessary but not sufficient.

Lack of vaccines was the least reported barrier to vaccination. The government has worked with organizations such as VillageReach over a period of many years to improve vaccine supply in both Maputo and Niassa,(8, 9, 18) so to some extent, this may be reflective of a positive impact from those efforts. The few cases of stock-outs were likely linked to national or even global stock-outs of BCG, rotavirus, and inactivated polio virus vaccines throughout the year that affected their availability at immunization posts.(19) What was striking, however, was that even when vaccines were available sometimes patients were turned away because it is the wrong day, or health worker could not confirm the eligibility of the child.

Also, physical accessibility of vaccination as a result of both long distances and poor transport infrastructure was a major barrier identified by both caregivers and health workers during FGDs. This challenge highlights a common issue related to transport required for health workers to conduct outreach services, which in many countries occurs less frequently than planned. This in turn means that many caregivers have to travel long distances to access health services. Therefore, extending the reach of health facilities, by putting plans in place to have more frequent outreach or mobile sessions, particularly in remote areas or in areas with vulnerable populations, must be prioritized.

It is critical to improve knowledge of vaccination among caregivers, health workers, and community leaders.

Our evidence demonstrated that both caregivers and health workers had suboptimal understanding related to immunization. During validation of dates recorded in HBR photos, we noted poor vaccination recording practices. This can lead to both health workers and caregivers not knowing if a child received

For peer review only - http://bmjopen.bmj.com/site/about/guidelines.xhtml

all due vaccines. To reduce MOV, caregivers need to understand the vaccination schedule, the value of vaccination, the diseases that each vaccine protects against, the importance of retaining the HBR, and potential AEFI and how to manage them. Given the low literacy levels among many of the caregivers, health facilities need materials and training that support information-sharing with low-literate populations. Health workers need to understand their own responsibility for both promoting and prompting vaccination as opportunities arise, and to be well-versed in the vaccination schedule. Engaging local radio and community leaders in sharing information about the importance of vaccination, and about specific issues related to immunization, can help create a more informed environment for both caregivers and health workers.

Health facilities need better strategies for managing data, electronic registration, and retention. Health workers, in all departments, need the ability to quickly and easily check the vaccination status of each eligible child who presents at the health facility. Currently, the majority of health facilities use paper-based immunization registers, which are difficult to access information and should be better designed (or health worker should be better trained on how to use them). Also, if caregivers always brought HBR to each health facility encounter and children have their HBR checked regularly, this could facilitate cross-referral, which could in turn greatly reduce MOV and likelihood of vaccine preventable disease morbidity and mortality. With continued advancement of electronic databases that prevent (or minimize) discrepancies in data, detect registry errors, and allow quick and easy queries, these could significantly increase the ability of health facilities to identify children who are due for vaccination, to follow children over the course of an immunization series, and to track and identify drop-outs. However, this would require significant investment for equipment, training, access to electricity and long-term maintenance. In the meantime, efforts to maximize the design and use of paper-based records should continue to be emphasized.

Limitations

As with past MOV assessments, (11-13, 15, 20) this assessment was not intended to be nationally representative or representative of each of the three provinces that were assessed. It should be interpreted as a programme assessment to identify areas which can be improved to reduce MOV. The assessment was carried out over a relatively short time period (10 to 12 days), in a small sample of health facilities and the estimation of MOV was limited only to children with documented vaccination dates extracted from children's HBRs or from health facility registers. As a result, other reasons for MOV may have been missed. In addition, through validation of vaccination documentation photos, we noted

For peer review only - http://bmjopen.bmj.com/site/about/guidelines.xhtml

poor vaccination recording practices in HBRs. This could have resulted in many children being under- or over-vaccinated with certain antigens, leading to over- or under-estimation of MOV.

CONCLUSION

What we learned in Mozambique confirms a growing global understanding that MOV continue to occur within health facilities. The WHO emphasizes that in order to reduce MOV, efforts should be made on a day-to-day basis at the health facility level to screen and refer children for vaccination. The results discussed in this report provide evidence and guidance for creating a roadmap for reducing MOV specific to Mozambique.

Our discussions with stakeholders at the province and national levels indicate a willingness and commitment to reduce MOV. The country has the potential to ensure supplies are available to all populations, improve the timeliness of vaccination services, improve the efficiency of health service delivery, and promote synergies between preventive and curative services at the health facility level. The leadership by provinces in developing tailored action plans and the interaction at national level are impressive. It is critical that funding and technical partners appropriately resource and support these plans with staff and financial resources. Immunization stakeholders in Mozambique should consider these data and continue to build the findings into country planning, resource, and accountability mechanisms, with the goal of maximizing every opportunity to ensure children are fully immunized.

Acknowledgments

The authors wish to thank the Ministry of Health (Ministério da Saúde de Moçambique (MISAU)) management and province staff, researchers from Eduardo Mondlane University and partners for supporting the MOV assessment. We also wish to thank the caregivers and health workers who took the time to participate in the MOV assessment. Finally, we would also like to acknowledge the input of Melissa West, (VillageReach) during the early phases of manuscript development and Stephanie Shendale (WHO) during the planning phases of the MOV assessment.

For peer review only - http://bmjopen.bmj.com/site/about/guidelines.xhtml

REFERENCES

1. Magadzire BP, Joao G, Shendale S, Ogbuanu IU. Reducing missed opportunities for vaccination in selected provinces of Mozambique: A study protocol. Gates Open Research. 2017;1(5).
2. Sridhar S, Maleq N, Guillermet E, Colombini A, Gessner B. A systematic literature review of missed opportunities for immunization in low- and middle-income countries. Vaccine. 2014;32(51):6870-9.
3. Hutchins SS, Jansen HA, Robertson SE, Evans P, Kim-Farley RJ. Studies of missed opportunities for immunization in developing and industrialized countries. 1993;71(5):549-60.
4. World Health Organization. Planning guide to reduce missed opportunities for vaccination (MOV) 2017 [Available from: http://apps.who.int/iris/bitstream/10665/259202/1/9789241512947-eng.pdf?ua=1.
5. World Health Organization. Methodology for the Assessment of Missed Opportunities for Vaccination 2017 [Available from: http://apps.who.int/iris/bitstream/10665/259201/1/9789241512954-eng.pdf?ua=1.
6. World Health Organization. Intervention guidebook for implementing and monitoring activities to reduce Missed Opportunities for Vaccination Geneva: World Health Organization; 2019 [Available from: https://www.who.int/immunization/documents/ISBN_9789241516310/en/.
7. World Health Organization, UNICEF. WHO and UNICEF estimates of national immunization coverage 2018 [Available from: http://apps.who.int/immunization_monitoring/globalsummary/timeseries/tswucoveragebcg.html.
8. Lee BY, Haidari LA, Prosser W, Connor DL, Bechtel R, Dipuve A, et al. Re-designing the Mozambique vaccine supply chain to improve access to vaccines. Vaccine. 2016;34(41):4998-5004.
9. Prosser W, Jaillard P, Assy E, Brown ST, Matsinhe G, Dekoun M, et al. System redesign of the immunization supply chain: Experiences from Benin and Mozambique. Vaccine. 2017;35(17):2162-6.
10. Burki T. Vaccine-derived poliovirus cases exceed wild types. The Lancet Infectious diseases. 2019;19(2):140.
11. Ogbuanu IU, Li AJ, Anya B-pM, Tamadji M, Chirwa G, Chiwaya KW, et al. Can vaccination coverage be improved by reducing missed opportunities for vaccination? Findings from assessments in Chad and Malawi using the new WHO methodology. PloS one. 2019;14(1):e0210648.
12. Kaboré L, Meda B, Médah I, Shendale S, Nic Lochlainn L, Sanderson C, et al. Assessment of missed opportunities for vaccination (MOV) in Burkina Faso using the World Health Organization's revised MOV strategy: Findings and strategic considerations to improve routine childhood immunization coverage. Vaccine. 2020.
13. Li AJ, Peiris TSR, Sanderson C, Nic Lochlainn L, Mausiry M, da Silva RBJBM, et al. Opportunities to improve vaccination coverage in a country with a fledgling health system: Findings from an assessment of missed opportunities for vaccination among health center attendees—Timor Leste, 2016. Vaccine. 2019;37(31):4281-90.
14. Li AJ, Tabu C, Shendale S, Okoth PO, Sergon K, Maree E, et al. Qualitative insights into reasons for missed opportunities for vaccination in Kenyan health facilities. PloS one. 2020;15(3):e0230783.
15. Li AJ, Tabu C, Shendale S, Sergon K, Okoth PO, Mugoya IK, et al. Assessment of missed opportunities for vaccination in Kenyan health facilities, 2016. PloS one. 2020;15(8):e0237913.
16. Biellik RJ, Davis R. The new World Health Organization recommendation on the 2-dose measles vaccine schedule and the way forward in African Region. Pan Afr Med J. 2017;27(Suppl 3):14.
17. Cambaza E, Mongo E, Anapakala E, Nhambire R, Singo J, Machava E. Outbreak of Cholera Due to Cyclone Kenneth in Northern Mozambique, 2019. International journal of environmental research and public health. 2019;16(16).
18. Kane K. Evaluation of the project to support PAV (Expanded Program on Immunization) in northern Mozambique; 20001-2008: an independent review for VillageReach with program and policy recommendations. 2008.
19. Lydon P, Raubenheimer T, Arnot-Krüger M, Zaffran M. Outsourcing vaccine logistics to the private sector: The evidence and lessons learned from the Western Cape Province in South-Africa. Vaccine. 2015;33(29):3429-34.
20. Olorunsaiye CZ, Langhamer MS, Wallace AS, Watkins ML. Missed opportunities and barriers for vaccination: a descriptive analysis of private and public health facilities in four African countries. Pan Afr Med J. 2017;27(Suppl 3):6.

Footnotes

Contributors: All authors conceptualized the study, LN and IUO developed the methodology, BM and GJ led data collection, BM and GJ conducted qualitative data analysis, LN conducted quantitative data analysis. All authors reviewed the results. BM developed the first draft manuscript; all authors reviewed and approved the manuscript.

For peer review only - http://bmjopen.bmj.com/site/about/guidelines.xhtml

Funding: The Bill and Melinda Gates Foundation (Grant number: OPP1146114) and the World Health Organization supported this study.

Competing interests: None declared.

Data availability statement: All data relevant to the study are included in the article or uploaded as supplementary information. Data are available upon reasonable request.

Figure 1. Flow chart for estimating missed vaccine opportunities (MOV) in three provinces in Mozambique.

For peer review only

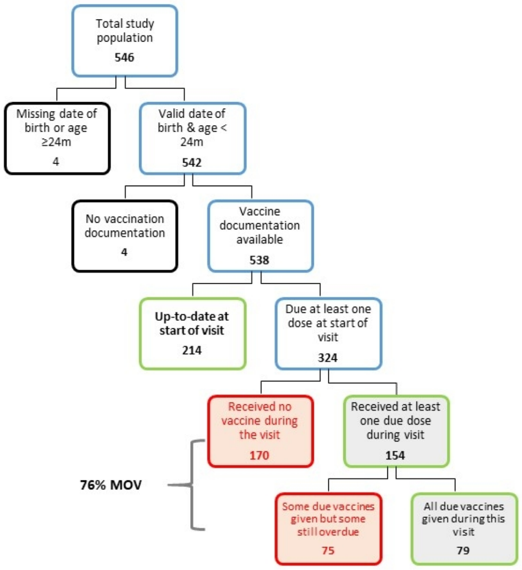

Figure 1. Flow chart for estimating missed vaccine opportunities (MOV) in three provinces in Mozambique.

190x338mm (96 x 96 DPI)

Supplementary Table 1. Immunization performance indicators among selected provinces and districts in Mozambique.

Province	District	Performance indicator*: Immunization coverage throughout province or district	Health facilities with functioning refrigerators	Health facilities experiencing a stockout of at least one antigen
Niassa	Mecanhelas	98%	78%	10%
	Majune	40%	50%	25%
Zambezia	Nicoadala	86%	85%	19%
	Gile	53%	50%	19%
Maputo Provincia	Manhica	83%	85%	13%
	Namahacha	35%	78%	19%

*Average for March through June 2017. (Source: Sistema Electrónico de Logística de Vacinas and District Health Information System 2 (SELV and DHIS2))

For peer review only

Supplementary Table 2: Time intervals used to classify timeliness of vaccination doses received using the nationally recommended ages for vaccination in Mozambique, 2017.

Vaccine	Recommended age of vaccination	Vaccination given; too early	Vaccination given; timely	Vaccination given: delayed
**Bacille Calmette-Guerin (BCG)**	At birth up to 23 months of age	--	0-45 days	>45 days
**Zero dose of OPV**	At birth or before 6 weeks of age	--	0-45 days	>45 days
**First dose of penta, OPV, PCV, and rotavirus**	At 2 months up to 23 months of age	<60 days	60-89 days	>89 days
**Second dose of penta, OPV, PCV, and rotavirus***	At 3 months or 4 weeks after first dose(s) up to 23 months of age*	<90 days	90-119 days	>119 days
**Third dose of penta, OPV, PCV, IPV and rotavirus***	At 4 months or 4 weeks after second dose(s) up to 23 months of age*	<120 days	120-149 days	>149 days
**First dose of measles**	9 months up to 23 months of age	<270 days	270-299 days	>299 days
**Second dose of measles and rubella**	18 months up to 23 months of age	<540 days	540-569 days	>569 days

Abbreviations: OPV, oral poliovirus vaccine; penta, diphtheria-tetanus-pertussis-hepatitis B–*Haemophilus influenzae* type b vaccine; IPV, inactivated poliovirus vaccine; PCV, pneumococcal conjugate vaccine.
* Rotavirus vaccine given up to 14 weeks of age.

For peer review only

Supplementary Table 3. Characteristics of surveyed caregivers of children with documented vaccination histories, Mozambique, 2017.

CAREGIVER SURVEY	n	%
	538	
Child demographics		
**Sex**		
Female	273	51
Male	265	49
**Age**		
<12 months	396	74
≥12 months	142	26
**Province**		
Maputo	193	36
Niassa	183	34
Zambezia	162	30
**Ever vaccinated**	525	
Yes	450	87
No	75	13
Caregiver demographics		
**Sex**	538	
Female	524	97
Male	14	3
**Relationship to child**	535	
Mother	508	95
Father	22	4
Uncle/aunt/grandparent	5	1
**Can read and write**	529	
Yes	236	45
No	293	55
**Educational Level**	532	
No educational qualifications	216	41
Did not complete primary education (less than 6 years)	108	20
Completed primary school	153	29
Completed secondary school or had higher education	55	10
Knowledge and attitudes		
**Child has home-based record**		
Yes, available at visit	533	99
Yes, but not available at visit	3	<1

For peer review only

No	2	<1
**Do you know precisely the vaccines that your child needs?**	515	
Yes	79	15
No	387	75
Not sure	49	10
**What is the purpose of a vaccines?***	535	
To prevent diseases	372	70
So children grow up healthy	219	41
To cure or heal diseases	28	5
They don't do any good	2	0
Not sure	112	21
Health facility visit		
**Type of health facility**	522	
Health center	500	96
Hospital	22	4
**Purpose of health facility visit**		
Medical consultation	145	27
Vaccination	143	27
Healthy child check-up	221	41
Accompanying caregiver	18	3
Child registration	7	1
Hospitalization	3	<1
**Health worker asked for the home-based record**	537	
Yes	464	86
No	73	14
**If vaccinated today, informed about next vaccination date?**	147	
Yes	113	77
No	34	23
**If vaccinated today, told which vaccines were given?**	147	
Yes	71	48
No	76	52
**If vaccinated today, told about vaccination reactions?**	147	
Yes	52	35
No	95	64
**If vaccinated today, satisfied with service?**	147	
Yes	141	96
No	6	4

For peer review only

**Suggestions for improving vaccination services***	530	
There should be more vaccination staff	120	23
There should be less waiting time	111	21
More information about vaccines given, diseases vaccines prevent, and AEFIs	93	18
There should always be vaccines in stock and functioning fridge	78	15
Friendlier treatment of the public	58	11
No suggestions or not sure	223	42

*Respondents allowed to select multiple responses.

For peer review only

Supplementary Table 4. Characteristics and knowledge, attitudes, and practices of surveyed health workers, Mozambique, 2017.

**HEALTH WORKER SURVEY**	n	%
	223	
Health worker demographics		
**Sex**	222	
Female	120	54
Male	102	46
**Professional training**	222	
Doctor	5	2
Nurse	91	41
Public employee in the health sector	9	4
Other	117	53
**Years of experience**	219	
0 to 4	144	66
5 to 9	66	30
10+	9	3
**Ever trained in vaccination or vaccine-preventable diseases**	221	
Yes	183	83
No	38	17
Health worker knowledge, attitudes, practices		
**My knowledge of vaccination and the EPI is sufficient to meet its needs**	222	
Agree	174	78
Disagree	48	22
**Contraindications for any vaccine***	219	
Local reaction to previous dose	60	27
Low grade fever	95	43
Seizures under medical treatment	142	65
Pneumonia and other serious diseases	71	32
None of the above	26	12
**When should vaccination status be assessed?***	222	
During child welfare visit	72	32
Consultation for any illness	61	27
When a child is accompanying a caregiver during a prenatal examination	17	8
When a child is accompanying a caregiver who visits a health centre for any reason	28	13

For peer review only

All of the above	112	50
**Why is vaccination status incomplete for some children?***	222	
Parents' negative beliefs related to vaccination	124	56
Hours of vaccination are incompatible with schedule of parents	28	13
Health workers do not review children's vaccination cards or about vaccination status	24	11
False contraindications for vaccination by health workers	36	16
Distance from vaccination site	174	78
All of the above	16	7
**I fear adverse reactions to vaccines**	220	
Agree	158	72
Disagree	62	28
**Completing nominal registers delays timely vaccination**	221	
Agree	44	20
Disagree	177	80
**There is sufficient staff offering immunization services at this facility**	49	
Agree	33	67
Disagree	16	33
**Enough vials of vaccine for all patients in need**	49	
Agree	38	78
Disagree	11	22

*Respondents allowed to select multiple responses.

For peer review only

STROBE 2007 (v4) Statement—Checklist of items that should be included in reports of *cross-sectional studies*

Section/Topic	Item #	Recommendation	Reported (Yes/No), page (p)
**Title and abstract**	1	(*a*) Indicate the study's design with a commonly used term in the title or the abstract	Yes p1
		(*b*) Provide in the abstract an informative and balanced summary of what was done and what was found	Yes p2
**Introduction**			
Background/rationale	2	Explain the scientific background and rationale for the investigation being reported	Yes p3-4
Objectives	3	State specific objectives, including any pre-specified hypotheses	Yes p4
**Methods**			
Study design	4	Present key elements of study design early in the paper	Yes p4
Setting	5	Describe the setting, locations, and relevant dates, including periods of recruitment, exposure, follow-up, and data collection	Yes p4-6
Participants	6	(*a*) Give the eligibility criteria, and the sources and methods of selection of participants	Yes p5
Variables	7	Clearly define all outcomes, exposures, predictors, potential confounders, and effect modifiers. Give diagnostic criteria, if applicable	Yes p9-13
Data sources/ measurement	8*	For each variable of interest, give sources of data and details of methods of assessment (measurement). Describe comparability of assessment methods if there is more than one group	Yes p9-13
Bias	9	Describe any efforts to address potential sources of bias	Yes p16
Study size	10	Explain how the study size was arrived at	Yes p5-6 (methodology has been fully described elsewhere)
Quantitative variables	11	Explain how quantitative variables were handled in the analyses. If applicable, describe which groupings were chosen and why	Yes p6
Statistical methods	12	(*a*) Describe all statistical methods, including those used to control for confounding	Yes p6-7
		(*b*) Describe any methods used to examine subgroups and interactions	Not Applicable
		(*c*) Explain how missing data were addressed	Yes p9

For peer review only

		(*d*) If applicable, describe analytical methods taking account of sampling strategy	Yes p6-7
		(*e*) Describe any sensitivity analyses	Not Applicable
**Results**			
Participants	13*	(a) Report numbers of individuals at each stage of study—e.g. numbers potentially eligible, examined for eligibility, confirmed eligible, included in the study, completing follow-up, and analysed	Yes (Figure 1)
		(b) Give reasons for non-participation at each stage	Yes (Figure 1)
		(c) Consider use of a flow diagram	Yes (Figure 1)
Descriptive data	14*	(a) Give characteristics of study participants (eg demographic, clinical, social) and information on exposures and potential confounders	Yes p9
		(b) Indicate number of participants with missing data for each variable of interest	Yes (Figure 1)
Outcome data	15*	Report numbers of outcome events or summary measures	Not applicable
Main results	16	(*a*) Give unadjusted estimates and, if applicable, confounder-adjusted estimates and their precision (eg, 95% confidence interval). Make clear which confounders were adjusted for and why they were included	Not applicable
		(*b*) Report category boundaries when continuous variables were categorized	Not applicable
		(*c*) If relevant, consider translating estimates of relative risk into absolute risk for a meaningful time period	Not applicable
Other analyses	17	Report other analyses done—eg analyses of subgroups and interactions, and sensitivity analyses	Not applicable
**Discussion**			
Key results	18	Summarise key results with reference to study objectives	Yes p14
Limitations	19	Discuss limitations of the study, taking into account sources of potential bias or imprecision. Discuss both direction and magnitude of any potential bias	Yes p16-17
Interpretation	20	Give a cautious overall interpretation of results considering objectives, limitations, multiplicity of analyses, results from similar studies, and other relevant evidence	Yes p14-18
Generalisability	21	Discuss the generalisability (external validity) of the study results	Yes p16-17
**Other information**			
Funding	22	Give the source of funding and the role of the funders for the present study and, if applicable, for the original study on which the present article is based	Yes p20

For peer review only

BMJ Open

Reducing missed opportunities for vaccination in Mozambique: Findings from a cross-sectional assessment conducted in 2017

Journal:	*BMJ Open*
Manuscript ID	bmjopen-2020-047297.R1
Article Type:	Original research
Date Submitted by the Author:	26-Aug-2021
Complete List of Authors:	Magadzire, Bvudzai; VillageReach, Global Technical Team<br>Joao, Gabriel; VillageReach<br>Bechtel, Ruth; VillageReach<br>Matsinhe, Graça; Ministro da Saude<br>Nic Lochlainn, Laura; World Health Organization<br>Ogbuanu, Ikechukwu Udo; World Health Organization
**Primary Subject Heading**:	Public health
Secondary Subject Heading:	Health services research
Keywords:	PUBLIC HEALTH, Paediatric infectious disease & immunisation < PAEDIATRICS, International health services < HEALTH SERVICES ADMINISTRATION & MANAGEMENT, PRIMARY CARE, Community child health < PAEDIATRICS

SCHOLARONE™
Manuscripts

I, the Submitting Author has the right to grant and does grant on behalf of all authors of the Work (as defined in the below author licence), an exclusive licence and/or a non-exclusive licence for contributions from authors who are: i) UK Crown employees; ii) where BMJ has agreed a CC-BY licence shall apply, and/or iii) in accordance with the terms applicable for US Federal Government officers or employees acting as part of their official duties; on a worldwide, perpetual, irrevocable, royalty-free basis to BMJ Publishing Group Ltd ("BMJ") its licensees and where the relevant Journal is co-owned by BMJ to the co-owners of the Journal, to publish the Work in this journal and any other BMJ products and to exploit all rights, as set out in our licence.

The Submitting Author accepts and understands that any supply made under these terms is made by BMJ to the Submitting Author unless you are acting as an employee on behalf of your employer or a postgraduate student of an affiliated institution which is paying any applicable article publishing charge ("APC") for Open Access articles. Where the Submitting Author wishes to make the Work available on an Open Access basis (and intends to pay the relevant APC), the terms of reuse of such Open Access shall be governed by a Creative Commons licence – details of these licences and which Creative Commons licence will apply to this Work are set out in our licence referred to above.

Other than as permitted in any relevant BMJ Author's Self Archiving Policies, I confirm this Work has not been accepted for publication elsewhere, is not being considered for publication elsewhere and does not duplicate material already published. I confirm all authors consent to publication of this Work and authorise the granting of this licence.

For peer review only

Title: Reducing missed opportunities for vaccination in Mozambique: findings from a cross-sectional assessment conducted in 2017

Short title: Reducing missed opportunities for vaccination in Mozambique

Authors:

Bvudzai Priscilla MAGADZIRE*, VillageReach, Global Technical Team (bvudzai.magadzire@villagereach.org), 2900 Eastlake Ave. E, Suite 230, Seattle, Washington 98102, USA

Gabriel JOAO, VillageReach Mozambique Country office (gabriel.joao@villagereach.org)

Ruth BECHTEL, VillageReach Mozambique Country office (ruth.bechtel@villagereach.org)

Graca MATSINHE, Ministério da Saúde de Moçambique, Expanded Program on Immunization, Maputo, Mozambique (gmatsinhe@gmail.com)

Laura NIC LOCHLAINN, World Health Organization, Department of Immunization, Vaccines and Biologicals, Geneva, Switzerland (niclochlainnl@who.int)

Ikechukwu Udo OGBUANU, World Health Organization, Department of Immunization, Vaccines and Biologicals, Geneva, Switzerland (dr_iyke@yahoo.com)

*Corresponding author

Keywords: missed opportunities for vaccination; immunization; vaccination coverage; health-care utilization; Mozambique

For peer review only

ABSTRACT

Objective: Missed opportunities for vaccination (MOV) are a significant contributor to low vaccination coverage. To better understand the magnitude and underlying causes of MOV among children aged 0–23 months in Mozambique, we conducted an assessment in 2017 and developed a roadmap for strengthening the country's childhood immunization programme.

Setting: Three provinces in North, South and Central Mozambique.

Methods: The assessment applied a mixed-methods approach. From a sample of 41 health facilities, we conducted exit interviews with caregivers of children aged 0–23 months (n=546), surveys with health workers (n=223), focus group discussions with caregivers (n=6) and health workers (n=5), and in-depth interviews with facility managers (n=9). We analysed the data to assess the magnitude of MOV and to identify causes of MOV and ways of preventing them.

Results: Vaccination records were available for 538 children. Sixty per cent (n=324) were eligible for vaccination on arriving for their health facility visit. Of these, 76% (n=245) were not fully vaccinated, constituting . Our analysis shows that these MOV were most frequently attributable to practices of caregivers, health workers and health systems reasons. Inadequate information about vaccination among both caregivers and health workers, poor or poorly understood health facility practices, inadequate integration of curative and preventative services, inadequate tracking systems to identify children due for vaccination and, less often, limited supply of vaccines, syringes and other related supplies at service points resulted in MOV.

Conclusions: The results of the assessment informed the development of roadmaps for reducing MOV that may be applicable to other settings. The global immunization community should continue to invest in efforts to reduce MOV and thereby make health service visits more effective and efficient for vaccination.

For peer review only

Strengths and limitations of this study

- Ministry of Health teams were able to use the results immediately to inform the development of roadmaps to reduce MOV.
- The assessment covered a relatively short time period (10–12 days) and a small sample of health facilities, and only included children with documented vaccinations. Consequently, other reasons for MOV may have been missed.
- As with past MOV assessments, the current assessment was not intended to be nationally representative or representative of any of the three provinces that were sampled. Results may therefore not be generalizable.
- MOV were estimated based only on children with documented vaccination dates extracted from the children's home-based records or from health facility registers. We noted poor vaccination recording practices that may have resulted in many children being under- or over-vaccinated with certain antigens, leading to over- or underestimation of MOV.

INTRODUCTION

Globally, the proportion of children receiving three diphtheria-tetanus-pertussis routine immunizations is below the global target of 90%, and has remained around 85% since 2015.(1) Although several factors may account for this gap, persistently high rates of missed opportunities for vaccination (MOV), particularly in low- and middle-income countries, are a significant contributor.(2) The World Health Organization (WHO) defines as "any contact with health services by an individual who is eligible for vaccination, which does not result in them receiving all the vaccine doses for which he or she is eligible".(3-5) It is widely assumed that children who are consistently missed by national immunization programmes have limited access to health services – one in five children by global estimates, hence a focus on reaching the "fifth child". Evidence suggests that a significant proportion of these unvaccinated children may already be accessing treatment and even seeking vaccination services, but they are still not receiving all of the recommended routine vaccinations.(3-5)

There is increasing momentum at the global, regional and country levels to address factors that may result in MOV. Accordingly, since 2015 WHO has been working with multiple immunization partners to improve and scale up its global MOV strategy. This strategy comprises four main components: the *Planning Guide to Reduce Missed Opportunities for Vaccination*,(4) the revised and updated *Methodology for the Assessment of Missed Opportunities for Vaccination*,(5) the *Intervention Guidebook for Implementing and Monitoring Activities to Reduce Missed Opportunities for Vaccination (MOV)*,(6)

For peer review only - http://bmjopen.bmj.com/site/about/guidelines.xhtml

and coordination among immunization partners and countries.

The MOV strategy aims to answer three essential questions:

- How many opportunities for vaccination are missed at existing vaccination sites?
- Why are opportunities for vaccination missed at these vaccination sites?
- What can be adjusted or done differently (e.g. changes in policies, changes in behaviour, or structural or organizational changes) to reduce MOV?

By identifying the underlying causes of MOV, countries will be able to tailor health system interventions to reduce MOV, with the goal of improving vaccine coverage, timeliness, and equity.

The Mozambique Expanded Programme on Immunization (EPI) faces challenges in increasing immunization coverage. For example, national coverage of the third dose of diphtheria-tetanus-pertussis and the first dose of measles-containing vaccine have remained at 80% and 85%, respectively, since 2015.(7) At the subnational level, many areas of the country have varying degrees of coverage. Approaches to strengthen vaccine delivery – from optimizing vaccine supply chains to short-term interventions such as immunization campaigns – also vary.(8, 9) Despite these multi-stakeholder efforts to address the challenges, new cases of circulating vaccine-derived poliovirus and pockets of unvaccinated children in Mozambique indicate an urgent need to strengthen the EPI.(10)

Consequently, in 2017 Mozambique's Ministry of Health, along with a consortium of partners that included WHO and VillageReach (a non-profit global health organization), implemented the MOV strategy in Mozambique.(4-6) The objectives were to estimate the prevalence of MOV the timeliness of vaccination; factors associated with MOV among children aged 0–23 months; and health-worker knowledge, attitudes and practices (KAP), and to formulate recommendations based on these data to improve immunization policies and practices in Mozambique.

METHODS

Study design and setting

The MOV assessment methodology uses a mixed-methods approach that incorporates both quantitative and qualitative tools which allows for triangulation of data. Quantitative data were collected by conducting exit interviews with caregivers and KAP surveys with health workers. In 2017, qualitative data were collected through focus group discussions (FGDs) with caregivers and health workers, as well as through in-depth interviews with health facility managers. The detailed MOV assessment methodology has been described elsewhere.(1, 11-15)

For peer review only - http://bmjopen.bmj.com/site/about/guidelines.xhtml

The Ministry of Health selected the following three provinces: Niassa (Northern region), Zambezia (Central region) and Maputo (Southern region) for geographic representation and because VillageReach is present in these regions. Two districts were selected in each province. We conducted a desk review using two electronic record systems as data sources: a system for vaccine logistics (SELV, Sistema Electrónico de Logística de Vacinas) and a monitoring and evaluation information system (SIS-MA, Sistema de Informação de Saúde para Monitoria e Avaliação). We reviewed data for a period of three months (April–June 2017), examining the following issues: administrative vaccination coverage, vaccine stock-out rates and functioning of cold-chain equipment (Supplementary Table 1). Districts with vaccination coverage greater than 80%, a stock-out rate lower than 10% and more than 95% of fridges functioning were considered “well performing”. Districts with vaccination coverage less than 50%, a stock-out rate higher than 10% and fewer than 95% of fridges functioning were considered “poor performing”.

Because health services are decentralized, within each province we selected a combination of comprehensive primary health-care and tertiary facilities that serve populations of different socioeconomic status in urban and rural areas.

Participant selection

Health facilities staff are made up of a mix of mostly nurses and technicians and a few doctors. For the health-worker KAP surveys and FGDs, we included health workers in the selected health facilities from both preventative and curative departments who were present on the day of the assessment.

Patient and public involvement

Caregivers collaborated with us as key informants. Their views and experiences alongside those of health workers shaped the design of proposed interventions. All caregivers aged at least 18 years exiting the health facility in the selected districts with a child that appeared to be between 0 and 23 months of age were eligible for inclusion in the exit surveys or FGDs. If more than one child accompanied a caregiver, the interviewers were instructed to select one child between 0 and 23 months of age.(5)

Data collection tools

The generic data collection tools in the WHO MOV methodology guide were translated into Portuguese.(5) All quantitative data were collected on password-protected tablets using survey forms uploaded to the survey software platform (Zegeba AS, Alesund, Norway). The data collectors directly

For peer review only - http://bmjopen.bmj.com/site/about/guidelines.xhtml

entered data from the vaccination documentation (home-based records or health facility registers) into the survey platform software, and a photo of the vaccination documentation was captured for data validation purposes. Qualitative data notes were recorded on paper and subsequently typed up and emailed to the MOV supervision team. Once paper notes were transcribed, and translated into English for analysis, they were destroyed.

Data collection and analysis

Prior to data collection, we conducted a week-long training with the field team, which comprised of four supervisors and eight data collectors from the Ministry of Health, independent researchers from Eduardo Mondlane University and VillageReach staff. To consolidate classroom training lessons and ensure comfort with electronic data entry of the surveys and photographing vaccination documentation, we piloted the data collection tools in nearby health facilities in Maputo City. Mozambique was the first country to use the Portuguese version of the WHO MOV tools; consequently, after the pilot, the team assessed the accuracy of the translation and amended the tools as necessary.

Field teams were deployed to the three provinces over a two-week period between October and November 2017. The teams aimed to achieve a daily team quota of 20 exit interviews with caregivers and 10 KAP surveys with health workers at each health facility. Exit interviews with caregivers were conducted in the morning, and health workers were interviewed in the afternoon. Health facility and provincial health officials were purposively selected to participate in in-depth interviews. Although an initial sample of 30 health facilities were selected centrally, team supervisors liaised with the central coordinator to add new sites if the session sizes at the health facility were insufficient to fulfil the daily team quota. Because data were captured primarily with electronic tools (tablets), quantitative data were uploaded daily to a central server and translated into English for analysis.

All quantitative analyses were conducted using Stata (StataCorp. 2015. Stata Statistical Software: Release 14. College Station, TX: StataCorp LP). Data analysis focused on estimating the extent of MOV and timeliness of vaccination using information collected from vaccination documentation. As part of the data cleaning process, all vaccination dates entered by the field teams during data collection were validated by reviewing each photo of the vaccination documentation. We further analysed other explanatory and demographic reasons for MOV, the classification of MOV by underlying cause (e.g. related to the health system, caregivers or health workers) and estimated the number of eligible doses missed, by antigen. We assessed the timeliness of vaccination doses of all vaccinations indicated on the

For peer review only

childrens' vaccination documents using the recorded date of birth, the dates of vaccination and the time intervals for vaccines recommended by the Mozambique EPI (Supplementary Table 2).

Using Atlas.ti (ATLAS.ti Scientific Software Development GmbH, version 8), we conducted a thematic analysis of the qualitative data transcripts to explore common, unique and conflicting themes, as well as to draw out illustrative quotes and key conclusions.

We shared the preliminary MOV assessment results with the leadership of the three provinces for verification and consideration to inform the development of future interventions to reduce MOV.

Ethics approval

The National Bioethics Committee in Mozambique (Comité Nacional de Bioética) granted ethical clearance for this assessment (Reference Number: 1008). In addition, each province (Niassa, Zambezia and Maputo) granted permission for the MOV assessment to be conducted. Before each interview, respondents were informed that participation was voluntary and optional. They were also informed that their responses would in no way affect their ability to access services or, in the case of health workers, threaten their employment.

RESULTS

Respondent characteristics

The field teams conducted the MOV assessment in 41 health facilities, visiting 11 more health facilities than planned due to the small session sizes encountered at some health facilities. The teams carried out exit interviews with 546 caregivers, of whom 98% (n=538) had children aged 0–23 months and 95% (n=521) had vaccination records. For 41% (n=221) of children aged 0–23 months, the purpose of the health facility visit was for a healthy child check-up, followed by 27% (n=145) for medical consultation; 27% (n=143) for vaccination; 3% (n=18) accompanying a caregiver; and 1% for child registration (n=7) or hospitalization (n=3) (Supplementary Table 3).

In terms of age, three-quarters (74%; n=396) of the children were younger than 12 months, and 51% (n=273) were female (Supplementary Table 3). The caregiver literacy rate (measured as reported ability to read and write) was 45% (n=236). Forty-one per cent (n=216) of caregivers had no educational qualifications, while 29% (n=153) had completed primary education and 10% (n=55) had completed secondary school or had higher education (Supplementary Table 3).

For peer review only - http://bmjopen.bmj.com/site/about/guidelines.xhtml

We also conducted KAP surveys with 223 health workers at the selected health facilities, including staff from preventative and curative service departments. The majority of health workers interviewed were either trained nurses (42%; n=93) or doctors (34%; n=75). The remainder were from other professional cadres, e.g. pharmacists or pharmacist technicians (7%; n=16) and laboratory technicians (4%; n=8). Among participating health workers across the three provinces, only 16% (n=49) routinely administer vaccines in EPI clinics. The remainder worked in outpatient departments or in oral health, general maternal and child health, family planning, nutrition or other specialty services (data not shown). Two-thirds (n=144) of health workers had fewer than four years of work experience. Most health workers (83%; n=183) reported they received training in vaccine-preventable diseases during basic pre-service professional training (Supplementary Table 4). Only 22% (n=48), however, had received additional in-service training. Of those who had received in-service training, 53% (n=24) had been trained in the past year, 20% (n=9) in the past two to three years and 27% (n=12) four or more years ago.

Finally, the field teams conducted six FGD sessions with caregivers, five FGD sessions with health workers and nine in-depth interviews with provincial health officials and health facility managers.

Frequency and causes of MOV

Proportion of children with MOV

The proportion of children with up-to-date vaccinations at the start of their health facility visit was 40% (n=214), including one child who was ineligible for further vaccination because of a reaction to a previous vaccine dose. Among the 324 children eligible for vaccination, 76% (n=245) had a MOV on the day of the assessment (Figure 1). Among the 324 children, 52% (n=170) received no vaccine, while 23% (n=75) received at least one dose during the visit, but were still due for other vaccines; 24% (n=79) received all due vaccines.

Figure 1. Flow chart for estimating missed vaccine opportunities (MOV) in three provinces in Mozambique, 2017.

Demographic and other factors correlated with MOV

We found the proportion of MOV differed slightly by sex, with 76% (n=118) of females compared to 70% (n=117) of males experiencing a MOV. Children aged 12 months or older had a higher proportion of MOV (95%; n=58) compared to children under 12 months (67%; n=177). Children who lived in Zambezia Province had a higher proportion of MOV (93%; n=98) compared to children from Maputo (76%; n=74) and Niassa (56%; n=61).

For peer review only - http://bmjopen.bmj.com/site/about/guidelines.xhtml

Forty-five per cent (n=59) of children eligible for vaccination who came to the health facility for vaccination had a MOV, compared with 91% (n=176) of children who were at the health facility for a non-vaccination visit. Among these children, 93% (n=69) had a MOV during their medical consultation, 89% (n=92) during a healthy child visit and 92% (n=12) when accompanying a caregiver.

When caregivers were asked why their child was not vaccinated during the health facility visit, 59% (n=92) said that the reason for their visit was not for vaccination or they believed their child was already fully vaccinated (18%; n=29). Caregivers also said that health workers told them their child was not eligible for vaccination (10%; n=15) or did not tell them about vaccination during the visit 8% (n=12) or that no vaccines were available in the health facility (8%; n=12) (Table 1).

For peer review only

Table 1. Numbers and proportion of missed opportunities for vaccination (MOV) by demographic and other factors in three provinces in Mozambique, 2017 (n=538).

	**Overall**	
	**No contraindications, and due at least 1 dose** n (%)	**MOV** n (%)
**Demographic or other factor**	324 (60%)	245 (76%)
Sex of child		
Female	156 (48%)	118 (76%)
Male	168 (52%)	117 (70%)
Age of child		
<12 months	263 (81%)	177 (67%)
≥12 months	61 (19%)	58 (95%)
Province		
Maputo	102 (31%)	76 (74%)
Niassa	114 (35%)	61 (56%)
Zambezia	105 (33%)	98 (93%)
Reason for visit		
Vaccination	130 (40%)	59 (45%)
Non-vaccination visit	194 (60%)	176 (91%)
i. Healthy child visit	*103 (32%)*	*92 (89%)*
ii. Medical consultation	*74 (23%)*	*69 (93%)*
iii. Accompanying caregiver	*12 (4%)*	*11 (92%)*
iv. Hospitalization	*2 (<1%)*	*2 (100%)*
v. Child registration	*2 (<1%)*	*2 (100%)*
Do you have a home-based record for this child?		
Yes, and I have it with me today	319 (98%)	230 (72%)
Yes, but I do not have it with me	3 (1%)	3 (100%)
No, I do not have a home-based record for this child	2 (1%)	2 (100%)
Reason not vaccinated today*		
Vaccination was not reason for visit	-	92 (59%)
Child is already fully vaccinated	-	29 (18%)
Health worker said child was not eligible for vaccination	-	15 (10%)
No vaccines in the health facility	-	12 (8%)
Health worker did not tell me about vaccinating child	-	6 (4%)
Other reasons	-	3 (2%)

**Respondents allowed to select multiple responses.*

Eligible vaccine doses missed, by antigen

Of the 851 eligible vaccine doses due, 53% were missed (n=447). Bacille Calmette-Guérin (BCG) (60%; n=40), first dose of oral poliovirus (OPV) (64%; n=39), third dose of OPV (66%; n=38), second dose of measles and rubella (89%; n=33) and inactivated poliovirus (IPV) vaccine (91% n=117) constituted the highest numbers of missed doses overall (Table 2).

For peer review only - http://bmjopen.bmj.com/site/about/guidelines.xhtml

Table 2. Missed opportunities by vaccine dose and timeliness of vaccine doses administered to surveyed children aged 0–23 months with documented vaccination history, Mozambique 2017.

Vaccine and dose	Eligible doses due n	Eligible due doses		Timeliness[1]			
		Given n *(%)*	Missed n (%)	Doses children received n	Vaccination given: too early %	Vaccination given: timely %	Vaccination given: delayed %
BCG	67	27 (40%)	40 (60%)	498	—	90%	10%
Zero dose OPV	32	20 (62%)	12 (38%)	461	—	97%	3%
First dose of penta	47	31 (66%)	16 (34%)	459	12%	76%	12%
First dose of OPV	61	22 (36%)	39 (64%)	436	12%	71%	17%
First dose of PCV	48	19 (60%)	29 (40%)	456	11%	76%	13%
First dose of rotavirus*	38	28 (74%)	10 (26%)	419	11%	75%	14%
Second dose of penta	47	39 (83%)	8 (17%)	417	9%	75%	16%
Second dose of OPV	45	25 (56%)	20 (44%)	385	9%	66%	25%
Second dose of PCV	48	38 (79%)	10 (21%)	411	9%	71%	20%
Second dose of rotavirus*	44	40 (91%)	4 (9%)	368	9%	50%	41%
Third dose of penta	49	25 (51%)	24 (49%)	350	6%	73%	21%
Third dose of OPV	58	20 (34%)	38 (66%)	321	6%	64%	29%
Third dose of PCV	55	26 (47%)	29 (53%)	340	6%	70%	24%
Inactivated poliovirus	129	12 (9%)	117 (91%)	274	16%	65%	18%
First dose of measles	46	28 (61%)	18 (39%)	216	14%	67%	19%
Second dose of measles and rubella	37	4 (11%)	33 (89%)	23	4%	39%	57%
**Total**	**851**	**404 (47%)**	**447 (53%)**	**5,834**	**10%**	**70%**	**21%**

Note: [1] Please see Supplementary Table 2 for time intervals used in classifying timeliness of vaccination as per the Mozambique immunization schedule. An em dash (—) indicates no eligible doses were due.
*Rotavirus vaccine given up to 14 weeks of age.
Abbreviations: BCG, Bacille Calmette-Guérin; OPV, oral poliovirus vaccine; penta, diphtheria-tetanus-pertussis-hepatitis B–*Haemophilus influenzae* type b vaccine; IPV, inactivated poliovirus vaccine; PCV, pneumococcal conjugate vaccine.

Timeliness of routine immunization

Based on analysis of all vaccinations indicated on vaccination documents, 70% (n=4,102) of the 5,834 documented vaccine doses were administered in a timely manner, while 10% (n=558) were too early and 21% (n=1,236) were delayed. The vaccine doses most commonly administered too early were IPV (16%; n=45) and the first dose of measles (14%; n=31) (Table 2). The most frequent delayed vaccines included the second dose of rotavirus (41%; n=151), the second dose of OPV (25%; n=98), the third dose of OPV (30%; n=94), and the second dose of measles and rubella (57%; n=13). Overall, the pentavalent and OPV vaccine series both showed decreasing timeliness, with doses due at older ages tending to be more delayed (Table 2).

For peer review only - http://bmjopen.bmj.com/site/about/guidelines.xhtml

We also found 13 children with more than one dose of IPV and 37 children with a third dose of rotavirus recorded in their home-based record (Supplementary Table 2).

Quality, availability, and accessibility of vaccines and vaccination services

During the health facility visit, 86% (n=464) of caregivers reported that the health worker asked to see their child's home-based record. Among the 154 caregivers who brought their child for vaccination on the day of the assessment, 96% (n=141) reported being satisfied with the services. However, 64% (n=95) were not told about vaccination reactions their child might have following vaccination, 52% (n=76) were not told what vaccines their child had been given, and 23% (n=32) were not informed about the next vaccination date (Supplementary Table 3).

Two-thirds (67%; n=33) of health workers who routinely administer vaccines in the EPI clinic felt there were sufficient staff to carry out vaccination activities. However, 22% (n=11) reported having insufficient supplies of specific vaccines, including for BCG, PCV, rotavirus and OPV vaccines (Supplementary Table 4). Other supplies that were reported to be lacking at the service delivery level included syringes, home-based records and safety boxes.

Physical accessibility (distances and geographic factors) to vaccination services was one of the major barriers identified by both caregivers and health workers in FGDs and in-depth interviews. Some caregivers stated that this is particularly challenging when the child is still very young; they did not want to expose them to harsh weather conditions, or they lacked transportation to the health facility:

> *What happens sometimes is that mothers do not appear…when it [health facility] is far away, they begin to have fear. Maybe they just come for those first vaccines until the [baby is] 4 months*. (Health worker, FGD, Zambezia Province)

Health workers referred to the important role of mobile brigades (outreach teams) in providing access to vaccination services to the communities. However, resources for mobile brigades, such as transportation and human resources, are limited. As a result, health workers said, maintaining a consistent outreach programme was challenging:

> *Another challenge is to cover the entire area of the administrative post including the absentees. There are many absentees. They may give the contact number, but it does not go through. Although some give their addresses, how do we get there? It's hard. Here, many times, we plan mobile brigades, but we can't run them because of insufficient transportation. We only have [an] ambulance and it is doing a lot of work hours and patient transfers*. (Health worker, FGD,

For peer review only - http://bmjopen.bmj.com/site/about/guidelines.xhtml

Maputo Province)

Knowledge and perceptions of vaccination among caregivers

Although a high percentage (99%; n=533) of caregivers with children aged 0–23 months had their child's home-based record on hand during the health facility visit, the majority of caregivers (75%; n=387) reported not knowing precisely what vaccines their child needed, and 21% (n=112) were unsure about the purpose of vaccination (Supplementary Table 3). Of the 78 caregivers who reported knowing the vaccines needed by their child, 36% (n=28) did not know when the vaccines should be given. Gaps in caregiver knowledge on issues related to vaccination were also emphasized in the FGDs:

> *We don't know all the vaccines. We know that they protect, yes, but each one protects what? Most of us don't know. Maybe an explanation in terms of what each of the vaccines do [would help]. And when they increase the vaccines, also explain to us why they are increasing and which vaccines they are adding.* (Caregiver, FGD, Niassa Province)

Overall, caregiver responses seemed to reflect that they placed high value on vaccination services; 92% (n=470) stated that children can get sick if they are not vaccinated, and 97% (n=507) reported never having lost a child's home-based record. However, caregivers also raised concerns in FGDs about vaccine acceptability. Caregivers mentioned that the number of vaccinations that children receive is too high:

> *The big problem is also the amount of vaccines. Too many vaccines at the same time. There should be a way to reduce the vaccines because children suffer. You see that a child of nine months gets four vaccines in a single day. That's a lot.* (Caregiver, FGD, Maputo Province)

Knowledge and perception of vaccination among health workers

Seventy-eight per cent (n=174) of health workers agreed that their knowledge about vaccines was outdated. A high percentage reported low-grade fever (43%; n=95) and seizures under medical treatment (63%; n=142) as contraindications for any vaccine. Seventy-two per cent (n=152) of health workers also reported that they feared adverse events following immunization. Only 63% (n=140) of health workers correctly selected all six vaccines that healthy children should receive. Fifty per cent (n=112) of health workers thought that a child's vaccination status should be checked during every type of health service encounter (Supplementary Table 4). More than one-third (37%; n=51) suggested that only the health professional responsible for vaccination has an obligation to ask about vaccination status. When health workers were asked why vaccination is incomplete for some children, 56% (n=124) reported that caregivers' negative perceptions of vaccination are a major contributing factor to children missing vaccinations.

For peer review only - http://bmjopen.bmj.com/site/about/guidelines.xhtml

Although 80% (n=177) of health workers did not perceive recording vaccine doses on health facility registers and home-based records as a cause of delayed vaccination (Supplementary Table 4), in FGDs they said that they needed improved systems to retrieve children's records quickly and follow up with children who did not return for vaccine doses:

> *Right now what we consider a challenge is actually the children's retention in the vaccination programme, from the first to the last shot. We have books that allow us to follow the child from day one in the same book. Now, the search for defaulters is one of the greatest challenges.* (Health worker, FGD, Maputo Province)

Health workers identified three actions as critical for follow-up of defaulting children: home visits, developing a weekly list of children with incomplete vaccinations and contacting caregivers by phone or other means. One of the three provinces was already implementing these interventions, but reported needing more resources to improve intervention effectiveness.

DISCUSSION

In this first assessment in Mozambique to assess MOV, we have shown that among 60% of children eligible and due for vaccinations at the start of a health facility visit, 76% had a MOV. This proportion of MOV is higher compared to other recent WHO led MOV assessments from Chad (51%), Malawi (66%), Timor Leste (41%), but similar in proportion to MOV assessments conducted in Kenya (75%) and Burkina Faso (76%) (11-13, 15, 20). Although 99% of caregivers had vaccination documentation available during the visit, which was the highest percentage observed in a MOV assessment to date, 9 in 10 children (91%) who were at the health facility for a non-vaccination visit had a MOV. We found that the proportion of MOV was higher among females (76%) compared to males (70%) and that 95% of children aged 12–24 months who were eligible for vaccination had a MOV. Nine in 10 children (93%) who lived in Zambezia Province had a MOV compared to 74% of children in Maputo and 56% of children in Niassa.

Evidence from a growing body of WHO-supported MOV assessments suggests a significant proportion of children with access to health services experience MOV.(3, 11) We found that even though children are accessing our health services, the health system continues to miss opportunities to vaccinate eligible children for a variety of reasons. In addition, more than nine out of ten caregivers whose child had a MOV said that the reason for the health facility visit was not for vaccination. This finding provides evidence that there is insufficient screening and referral of children eligible for vaccination. As such, it

For peer review only - http://bmjopen.bmj.com/site/about/guidelines.xhtml

highlights a need for improved integration between preventative and curative services. The other most significant factors for missing eligible children included a lack of adequate systems and practices for tracking children's vaccination status at the health facility level, inadequate caregiver knowledge and, less often, vaccine stock-outs or being refused services for coming on the wrong day.

Having a vaccination schedule that includes vaccines in the second year of life can often lead to a reduction in MOV, as it provides another opportunity for a child to receive catch-up vaccinations. In 2015 Mozambique introduced a second dose of measles and rubella at 18 months.(16) However, we found that almost all children aged 12–24 months who were eligible for vaccination had a MOV. In order to benefit from this contact point, the delivery platform for vaccines in the second year of life must be further strengthened in Mozambique.

During brainstorming and debriefing sessions at the provincial and national levels with EPI staff and immunization stakeholders, the key results were linked with actionable steps to reduce MOV and improve the EPI overall. Stakeholders deliberated on interventions that were, in their view, feasible in the short term. Provinces drafted action plans for reducing MOV as part of the 2019 EPI plans. Some of the themes that emerged during the brainstorming sessions follow. However, it must be noted that implementation of these plans was affected by Cyclone Idai and Cyclone Kenneth in early 2019, which had devastating consequences for Mozambique.(17)

- **Having available supplies in health facilities is necessary but not sufficient**

Lack of vaccines was the least reported barrier to vaccination. The government has worked with organizations such as VillageReach over many years to improve vaccine supply in both Maputo and Niassa,(8, 9, 18), so to some extent this finding reflects the positive impact of those efforts. The few cases of stock-outs were likely linked to national or even global stock-outs of BCG, rotavirus and IPV vaccines throughout the year, which affected their availability at EPI clinics.(19) It was striking, however, that even when vaccines were available, patients were sometimes turned away because they came on the wrong day, or a health worker could not confirm the child's eligibility.

- **Distance and transport limit access**

Also, access to vaccination due to long distances and poor transport infrastructure was a major barrier identified by both caregivers and health workers during FGDs. Transport is doubly challenging. It limits the ability of health workers to provide outreach services, which in many countries cannot be implemented as planned. It also limits access for many caregivers who must bring children over long distances to reach health services. Extending the reach of health facilities by implementing plans for more frequent outreach or mobile services, particularly in

For peer review only - http://bmjopen.bmj.com/site/about/guidelines.xhtml

remote areas or in areas with vulnerable populations, must therefore be prioritized.

- **Knowledge of vaccination among caregivers, health workers and community leaders must be improved**

Our evidence shows that both caregivers and health workers have suboptimal understanding of recommended immunization policies and practices. During validation of dates based on photos of home-based records, we noted poor vaccination recording practices. Consequently, both health workers and caregivers do not know whether a child has received all due vaccines. To reduce MOV, caregivers need to understand the diseases that each vaccine protects against, the vaccination schedule, the value of vaccination, the importance of retaining the home-based record, and potential adverse events following immunization and how to manage them. Given the low level of literacy among many caregivers, health facilities need materials and training appropriate for improving awareness through relevant information despite limited literacy. Health workers need to understand their own responsibility for both promoting vaccination and identifying available opportunities, based on practical knowledge of the vaccination schedule. Engaging local radio and community leaders in disseminating information about the importance of vaccination and about relevant aspects of immunization will help create a more informed environment for both caregivers and health workers.

- **Health facilities need better strategies for managing data, electronic registration and retention**

Health workers in both preventive and curative service departments should be able to quickly and easily check the vaccination eligibility and status of each child who presents at the health facility. Most health facilities currently use paper-based immunization registers, from which it is difficult to access information. Well-designed digital registers are needed, and health workers should be better trained to use them effectively. Techniques for designing and managing electronic databases can now minimize errors in entering data, detect and enable correction of discrepancies, and facilitate quick and easy queries, so that health facilities can identify children who are due for vaccination. Aims of effective health data monitoring include tracking children over the course of an immunization schedule and identifying dropouts. However, this technology would require significant investment for equipment, training, electricity infrastructure and long-term maintenance. In the meantime, efforts to maximize the design and use of paper-based records must continue to be emphasized. Furthermore, ensuring that caregivers always bring home-based records to each health facility visit and regularly checking children's home-based records would facilitate cross-checking clinical and home-based records. Implementing such practices should in turn greatly reduce MOV and the morbidity and mortality of vaccine-preventable diseases.

For peer review only - http://bmjopen.bmj.com/site/about/guidelines.xhtml

Limitations

As with past MOV assessments,(11-13, 15, 20), this study was not intended to be nationally representative or representative of any of the three provinces that were assessed. It should be regarded, however, as a programme assessment to identify areas which can be improved to reduce MOV. The assessment was carried out over a relatively short time period (10–12 days), in a small sample of health facilities. The estimation of MOV was limited to children with documented vaccination dates extracted from children's home-based records or from health facility registers. As a result, other reasons for MOV may have been missed. In addition, by validating vaccination documentation, we noted poor vaccination recording practices in home-based records. These practices could have resulted in many children being under- or over-vaccinated with certain antigens, leading to over- or underestimation of MOV.

CONCLUSION

What we learned in Mozambique confirms a growing global understanding that MOV continue to occur within health facilities. WHO emphasizes that in order to reduce MOV, diligent efforts are required in day-to-day practices of health facilities to screen and refer children for vaccination. The results discussed in this report provide evidence and guidance for creating a roadmap for reducing MOV in Mozambique. Our discussions with stakeholders at the provincial and national levels indicate a willingness and commitment to reduce MOV. The country now needs to realize its potential to ensure supplies are available to all populations, improve the timeliness of vaccination services, improve the efficiency of health service delivery, and promote synergies between preventive and curative services at the health facility level.

The leadership by provinces in developing tailored action plans and interactions at the national level are impressive. But funding and staff support for enhancing technical expertise and partners are critical. Based on our findings, immunization stakeholders in Mozambique should proceed with planning and action with the goal of maximizing every opportunity to ensure children are fully immunized.

Acknowledgements

The authors wish to thank the Ministry of Health (Ministerium da Saudi de Moçambique) management and province staff, researchers from Eduardo Mondlane University and partners for supporting the MOV assessment. We also wish to thank the caregivers and health workers who took the time to participate in the MOV assessment. Finally, we would also like to acknowledge the input of Melissa West

For peer review only - http://bmjopen.bmj.com/site/about/guidelines.xhtml

(VillageReach) during the early phases of manuscript development and Stephanie Shendale (WHO) during the planning phases of the MOV assessment.

Footnotes

Contributors: BPM, GJ, RB, GM, LNL, IUO conceptualized the study, LNL and IUO developed the methodology, BPM and GJ led data collection, BPM and GJ conducted qualitative data analysis and LNL conducted quantitative data analysis. BPM, GJ, RB, GM, LNL, IUO reviewed and validated the results. BPM developed the first-draft manuscript all authors reviewed and approved the manuscript.

Funding: The Bill and Melinda Gates Foundation (OPP1146114) and the World Health Organization supported this study.

Competing interests: None declared.

Data availability statement: All data relevant to the study are included in the article or uploaded as supplementary information. Data are available upon reasonable request.

For peer review only

REFERENCES

1. Magadzirefclinic BP, Joao G, Shendale S, Ogbuanu IU. Reducing missed opportunities for vaccination in selected provinces of Mozambique: A study protocol. Gates Open Research. 2017;1(5).
2. Sridhar S, Maleq N, Guillermet E, Colombini A, Gessner B. A systematic literature review of missed opportunities for immunization in low- and middle-income countries. Vaccine. 2014;32(51):6870-9.
3. Hutchins SS, Jansen HA, Robertson SE, Evans P, Kim-Farley RJ. Studies of missed opportunities for immunization in developing and industrialized countries. 1993;71(5):549-60.
4. World Health Organization. Planning guide to reduce missed opportunities for vaccination (MOV) 2017 [Available from: http://apps.who.int/iris/bitstream/10665/259202/1/9789241512947-eng.pdf?ua=1.
5. World Health Organization. Methodology for the Assessment of Missed Opportunities for Vaccination 2017 [Available from: http://apps.who.int/iris/bitstream/10665/259201/1/9789241512954-eng.pdf?ua=1.
6. World Health Organization. Intervention guidebook for implementing and monitoring activities to reduce Missed Opportunities for Vaccination Geneva: World Health Organization; 2019 [Available from: https://www.who.int/immunization/documents/ISBN_9789241516310/en/.
7. World Health Organization, UNICEF. WHO and UNICEF estimates of national immunization coverage 2018 [Available from: http://apps.who.int/immunization_monitoring/globalsummary/timeseries/tswucoveragebcg.html.
8. Lee BY, Haidari LA, Prosser W, Connor DL, Bechtel R, Dipuve A, et al. Re-designing the Mozambique vaccine supply chain to improve access to vaccines. Vaccine. 2016;34(41):4998-5004.
9. Prosser W, Jaillard P, Assy E, Brown ST, Matsinhe G, Dekoun M, et al. System redesign of the immunization supply chain: Experiences from Benin and Mozambique. Vaccine. 2017;35(17):2162-6.
10. Burki T. Vaccine-derived poliovirus cases exceed wild types. The Lancet Infectious diseases. 2019;19(2):140.
11. Ogbuanu IU, Li AJ, Anya B-pM, Tamadji M, Chirwa G, Chiwaya KW, et al. Can vaccination coverage be improved by reducing missed opportunities for vaccination? Findings from assessments in Chad and Malawi using the new WHO methodology. PloS one. 2019;14(1):e0210648.
12. Kaboré L, Meda B, Médah I, Shendale S, Nic Lochlainn L, Sanderson C, et al. Assessment of missed opportunities for vaccination (MOV) in Burkina Faso using the World Health Organization's revised MOV strategy: Findings and strategic considerations to improve routine childhood immunization coverage. Vaccine. 2020.
13. Li AJ, Peiris TSR, Sanderson C, Nic Lochlainn L, Mausiry M, da Silva RBJBM, et al. Opportunities to improve vaccination coverage in a country with a fledgling health system: Findings from an assessment of missed opportunities for vaccination among health center attendees—Timor Leste, 2016. Vaccine. 2019;37(31):4281-90.
14. Li AJ, Tabu C, Shendale S, Okoth PO, Sergon K, Maree E, et al. Qualitative insights into reasons for missed opportunities for vaccination in Kenyan health facilities. PloS one. 2020;15(3):e0230783.
15. Li AJ, Tabu C, Shendale S, Sergon K, Okoth PO, Mugoya IK, et al. Assessment of missed opportunities for vaccination in Kenyan health facilities, 2016. PloS one. 2020;15(8):e0237913.
16. Biellik RJ, Davis R. The new World Health Organization recommendation on the 2-dose measles vaccine schedule and the way forward in African Region. Pan Afr Med J. 2017;27(Suppl 3):14.
17. Cambaza E, Mongo E, Anapakala E, Nhambire R, Singo J, Machava E. Outbreak of Cholera Due to Cyclone Kenneth in Northern Mozambique, 2019. International journal of environmental research and public health. 2019;16(16).
18. Kane K. Evaluation of the project to support PAV (Expanded Program on Immunization) in northern Mozambique; 20001-2008: an independent review for VillageReach with program and policy recommendations. 2008.
19. Lydon P, Raubenheimer T, Arnot-Krüger M, Zaffran M. Outsourcing vaccine logistics to the private sector: The evidence and lessons learned from the Western Cape Province in South-Africa. Vaccine. 2015;33(29):3429-34.
20. Olorunsaiye CZ, Langhamer MS, Wallace AS, Watkins ML. Missed opportunities and barriers for vaccination: a descriptive analysis of private and public health facilities in four African countries. Pan Afr Med J. 2017;27(Suppl 3):6.

For peer review only - http://bmjopen.bmj.com/site/about/guidelines.xhtml

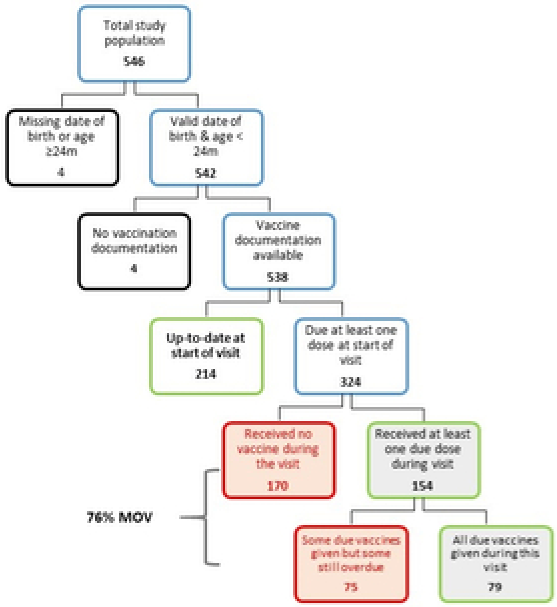

Figure 1. Flow chart for estimating missed vaccine opportunities (MOV) in three provinces in Mozambique.

30x28mm (300 x 300 DPI)

Supplementary Table 1. Immunization performance indicators among selected provinces and districts in Mozambique.

Province	District	Performance indicator*		
		Immunization coverage throughout province or district	Health facilities with functioning refrigerators	Health facilities experiencing a stockout of at least one antigen
Niassa	Mecanhelas	98%	78%	10%
	Majune	40%	50%	25%
Zambezia	Nicoadala	86%	85%	19%
	Gile	53%	50%	19%
Maputo Provincia	Manhica	83%	85%	13%
	Namahacha	35%	78%	19%

*Average for March through June 2017. (Source: Sistema Electrónico de Logística de Vacinas and District Health Information System 2 (SELV and DHIS2))

For peer review only

Supplementary Table 2: Time intervals used to classify timeliness of vaccination doses received using the nationally recommended ages for vaccination in Mozambique, 2017.

Vaccine	Recommended age of vaccination	Vaccination given; too early	Vaccination given; timely	Vaccination given: delayed
**Bacille Calmette-Guerin (BCG)**	At birth up to 23 months of age	--	0-45 days	>45 days
**Zero dose of OPV**	At birth or before 6 weeks of age	--	0-45 days	>45 days
**First dose of penta, OPV, PCV, and rotavirus**	At 2 months up to 23 months of age	<60 days	60-89 days	>89 days
**Second dose of penta, OPV, PCV, and rotavirus***	At 3 months or 4 weeks after first dose(s) up to 23 months of age*	<90 days	90-119 days	>119 days
**Third dose of penta, OPV, PCV, IPV and rotavirus***	At 4 months or 4 weeks after second dose(s) up to 23 months of age*	<120 days	120-149 days	>149 days
**First dose of measles**	9 months up to 23 months of age	<270 days	270-299 days	>299 days
**Second dose of measles and rubella**	18 months up to 23 months of age	<540 days	540-569 days	>569 days

Abbreviations: OPV, oral poliovirus vaccine; penta, diphtheria-tetanus-pertussis-hepatitis B–*Haemophilus influenzae* type b vaccine; IPV, inactivated poliovirus vaccine; PCV, pneumococcal conjugate vaccine.
* Rotavirus vaccine given up to 14 weeks of age.

For peer review only

Supplementary Table 3. Characteristics of surveyed caregivers of children with documented vaccination histories, Mozambique, 2017.

CAREGIVER SURVEY	n	%
	538	
Child demographics		
**Sex**		
Female	273	51
Male	265	49
**Age**		
<12 months	396	74
≥12 months	142	26
**Province**		
Maputo	193	36
Niassa	183	34
Zambezia	162	30
**Ever vaccinated**	525	
Yes	450	87
No	75	13
Caregiver demographics		
**Sex**	538	
Female	524	97
Male	14	3
**Relationship to child**	535	
Mother	508	95
Father	22	4
Uncle/aunt/grandparent	5	1
**Can read and write**	529	
Yes	236	45
No	293	55
**Educational Level**	532	
No educational qualifications	216	41
Did not complete primary education (less than 6 years)	108	20
Completed primary school	153	29
Completed secondary school or had higher education	55	10
Knowledge and attitudes		
**Child has home-based record**		
Yes, available at visit	533	99
Yes, but not available at visit	3	<1

For peer review only

No	2	<1
**Do you know precisely the vaccines that your child needs?**	515	
Yes	79	15
No	387	75
Not sure	49	10
**What is the purpose of a vaccines?***	535	
To prevent diseases	372	70
So children grow up healthy	219	41
To cure or heal diseases	28	5
They don't do any good	2	0
Not sure	112	21
Health facility visit		
**Type of health facility**	522	
Health center	500	96
Hospital	22	4
**Purpose of health facility visit**		
Medical consultation	145	27
Vaccination	143	27
Healthy child check-up	221	41
Accompanying caregiver	18	3
Child registration	7	1
Hospitalization	3	<1
**Health worker asked for the home-based record**	537	
Yes	464	86
No	73	14
**If vaccinated today, informed about next vaccination date?**	147	
Yes	113	77
No	34	23
**If vaccinated today, told which vaccines were given?**	147	
Yes	71	48
No	76	52
**If vaccinated today, told about vaccination reactions?**	147	
Yes	52	35
No	95	64
**If vaccinated today, satisfied with service?**	147	
Yes	141	96
No	6	4

**Suggestions for improving vaccination services***	530	
There should be more vaccination staff	120	23
There should be less waiting time	111	21
More information about vaccines given, diseases vaccines prevent, and AEFIs	93	18
There should always be vaccines in stock and functioning fridge	78	15
Friendlier treatment of the public	58	11
No suggestions or not sure	223	42

*Respondents allowed to select multiple responses.

For peer review only

Supplementary Table 4. Characteristics and knowledge, attitudes, and practices of surveyed health workers, Mozambique, 2017.

**HEALTH WORKER SURVEY**	n	%
	223	
Health worker demographics		
**Sex**	222	
Female	120	54
Male	102	46
**Professional training**	222	
Doctor	5	2
Nurse	91	41
Public employee in the health sector	9	4
Other	117	53
**Years of experience**	219	
0 to 4	144	66
5 to 9	66	30
10+	9	3
**Ever trained in vaccination or vaccine-preventable diseases**	221	
Yes	183	83
No	38	17
Health worker knowledge, attitudes, practices		
**My knowledge of vaccination and the EPI is sufficient to meet its needs**	222	
Agree	174	78
Disagree	48	22
**Contraindications for any vaccine***	219	
Local reaction to previous dose	60	27
Low grade fever	95	43
Seizures under medical treatment	142	65
Pneumonia and other serious diseases	71	32
None of the above	26	12
**When should vaccination status be assessed?***	222	
During child welfare visit	72	32
Consultation for any illness	61	27
When a child is accompanying a caregiver during a prenatal examination	17	8
When a child is accompanying a caregiver who visits a health centre for any reason	28	13

For peer review only

All of the above	112	50
**Why is vaccination status incomplete for some children?***	222	
Parents' negative beliefs related to vaccination	124	56
Hours of vaccination are incompatible with schedule of parents	28	13
Health workers do not review children's vaccination cards or about vaccination status	24	11
False contraindications for vaccination by health workers	36	16
Distance from vaccination site	174	78
All of the above	16	7
**I fear adverse reactions to vaccines**	220	
Agree	158	72
Disagree	62	28
**Completing nominal registers delays timely vaccination**	221	
Agree	44	20
Disagree	177	80
**There is sufficient staff offering immunization services at this facility**	49	
Agree	33	67
Disagree	16	33
**Enough vials of vaccine for all patients in need**	49	
Agree	38	78
Disagree	11	22

*Respondents allowed to select multiple responses.

For peer review only

STROBE 2007 (v4) Statement—Checklist of items that should be included in reports of *cross-sectional studies*

Section/Topic	Item #	Recommendation	Reported (Yes/No), page (p)
**Title and abstract**	1	(*a*) Indicate the study's design with a commonly used term in the title or the abstract	Yes p1
		(*b*) Provide in the abstract an informative and balanced summary of what was done and what was found	Yes p2
**Introduction**			
Background/rationale	2	Explain the scientific background and rationale for the investigation being reported	Yes p3-4
Objectives	3	State specific objectives, including any pre-specified hypotheses	Yes p4
**Methods**			
Study design	4	Present key elements of study design early in the paper	Yes p4
Setting	5	Describe the setting, locations, and relevant dates, including periods of recruitment, exposure, follow-up, and data collection	Yes p4-6
Participants	6	(*a*) Give the eligibility criteria, and the sources and methods of selection of participants	Yes p5
Variables	7	Clearly define all outcomes, exposures, predictors, potential confounders, and effect modifiers. Give diagnostic criteria, if applicable	Yes p9-13
Data sources/ measurement	8*	For each variable of interest, give sources of data and details of methods of assessment (measurement). Describe comparability of assessment methods if there is more than one group	Yes p9-13
Bias	9	Describe any efforts to address potential sources of bias	Yes p16
Study size	10	Explain how the study size was arrived at	Yes p5-6 (methodology has been fully described elsewhere)
Quantitative variables	11	Explain how quantitative variables were handled in the analyses. If applicable, describe which groupings were chosen and why	Yes p6
Statistical methods	12	(*a*) Describe all statistical methods, including those used to control for confounding	Yes p6-7
		(*b*) Describe any methods used to examine subgroups and interactions	Not Applicable
		(*c*) Explain how missing data were addressed	Yes p9

For peer review only

		(*d*) If applicable, describe analytical methods taking account of sampling strategy	Yes p6-7
		(*e*) Describe any sensitivity analyses	Not Applicable
**Results**			
Participants	13*	(a) Report numbers of individuals at each stage of study—e.g. numbers potentially eligible, examined for eligibility, confirmed eligible, included in the study, completing follow-up, and analysed	Yes (Figure 1)
		(b) Give reasons for non-participation at each stage	Yes (Figure 1)
		(c) Consider use of a flow diagram	Yes (Figure 1)
Descriptive data	14*	(a) Give characteristics of study participants (eg demographic, clinical, social) and information on exposures and potential confounders	Yes p9
		(b) Indicate number of participants with missing data for each variable of interest	Yes (Figure 1)
Outcome data	15*	Report numbers of outcome events or summary measures	Not applicable
Main results	16	(*a*) Give unadjusted estimates and, if applicable, confounder-adjusted estimates and their precision (eg, 95% confidence interval). Make clear which confounders were adjusted for and why they were included	Not applicable
		(*b*) Report category boundaries when continuous variables were categorized	Not applicable
		(*c*) If relevant, consider translating estimates of relative risk into absolute risk for a meaningful time period	Not applicable
Other analyses	17	Report other analyses done—eg analyses of subgroups and interactions, and sensitivity analyses	Not applicable
**Discussion**			
Key results	18	Summarise key results with reference to study objectives	Yes p14
Limitations	19	Discuss limitations of the study, taking into account sources of potential bias or imprecision. Discuss both direction and magnitude of any potential bias	Yes p16-17
Interpretation	20	Give a cautious overall interpretation of results considering objectives, limitations, multiplicity of analyses, results from similar studies, and other relevant evidence	Yes p14-18
Generalisability	21	Discuss the generalisability (external validity) of the study results	Yes p16-17
**Other information**			
Funding	22	Give the source of funding and the role of the funders for the present study and, if applicable, for the original study on which the present article is based	Yes p20

For peer review only
